# Structural genomic variation and behavioral interactions underpin a balanced sexual mimicry polymorphism

**DOI:** 10.1016/j.cub.2024.08.053

**Published:** 2024-09-25

**Authors:** Tristram O. Dodge, Bernard Y. Kim, John J. Baczenas, Shreya M. Banerjee, Theresa R. Gunn, Alex E. Donny, Lyle A. Given, Andreas R. Rice, Sophia K. Haase Cox, M. Luke Weinstein, Ryan Cross, Benjamin M. Moran, Kate Haber, Nadia B. Haghani, Jose Angel Machin Kairuz, Hannah R. Gellert, Kang Du, Stepfanie M. Aguillon, M. Scarlett Tudor, Carla Gutiérrez-Rodríguez, Oscar Rios-Cardenas, Molly R. Morris, Manfred Schartl, Daniel L. Powell, Molly Schumer

**Affiliations:** 1 Department of Biology, Stanford University, 327 Campus Drive, Stanford, CA 94305, USA; 2 Centro de Investigaciones Científicas de las Huastecas “Aguazarca” A.C., 16 de Septiembre, 392 Barrio Aguazarca, Calnali, Hidalgo 43240, México; 3 Center for Population Biology and Department of Evolution and Ecology, University of California, Davis, 475 Storer Mall, Davis, CA 95616, USA; 4 Department of Biological Sciences, Ohio University, 7 Depot St., Athens, OH 45701, USA; 5 Berkeley High School, 1980 Allston Way, Berkeley, CA 94704, USA; 6 Xiphophorus Genetic Stock Center, Texas State University, San Marcos, 601 University Drive, San Marcos, TX 78666, USA; 7 Department of Ecology and Evolutionary Biology, University of California, Los Angeles, 612 Charles E. Young Drive South, Los Angeles, CA 90095, USA; 8 Cooperative Extension and Aquaculture Research Institute, University of Maine, 33 Salmon Farm Road, Franklin, ME 04634, USA; 9 Red de Biología Evolutiva, Instituto de Ecología, A.C., Carretera antigua a Coatepec 351, Col. El Haya, Xalapa, Veracruz 91073, México; 10 Developmental Biochemistry, Biocenter, University of Würzburg, Am Hubland, 97074 Wuerzburg, Germany; 11 Department of Biology, Louisiana State University, 202 Life Science Building, Baton Rouge, LA 70803, USA; 12 Howard Hughes Medical Institute, 327 Campus Drive, Stanford, CA 94305, USA; 13 These authors contributed equally; 14 Lead contact

## Abstract

How phenotypic diversity originates and persists within populations are classic puzzles in evolutionary biology. While balanced polymorphisms segregate within many species, it remains rare for both the genetic basis and the selective forces to be known, leading to an incomplete understanding of many classes of traits under balancing selection. Here, we uncover the genetic architecture of a balanced sexual mimicry polymorphism and identify behavioral mechanisms that may be involved in its maintenance in the swordtail fish *Xiphophorus birchmanni*. We find that ~40% of *X. birchmanni* males develop a “false gravid spot,” a melanic pigmentation pattern that mimics the “pregnancy spot” associated with sexual maturity in female livebearing fish. Using genome-wide association mapping, we detect a single intergenic region associated with variation in the false gravid spot phenotype, which is upstream of *kitlga*, a melanophore patterning gene. By performing long-read sequencing within and across populations, we identify complex structural rearrangements between alternate alleles at this locus. The false gravid spot haplotype drives increased allele-specific expression of *kitlga*, which provides a mechanistic explanation for the increased melanophore abundance that causes the spot. By studying social interactions in the laboratory and in nature, we find that males with the false gravid spot experience less aggression; however, they also receive increased attention from other males and are disdained by females. These behavioral interactions may contribute to the maintenance of this phenotypic polymorphism in natural populations. We speculate that structural variants affecting gene regulation may be an underappreciated driver of balanced polymorphisms across diverse species.

## In brief

The false gravid spot is a sexual mimicry polymorphism that allows males to mimic females. Dodge et al. link the phenotype to structural variation that increases expression of the nearby *kitlga* gene. The spot affects multiple social interactions with males and females, which may contribute to the persistence of this balanced polymorphism in nature.

## Graphical Abstract

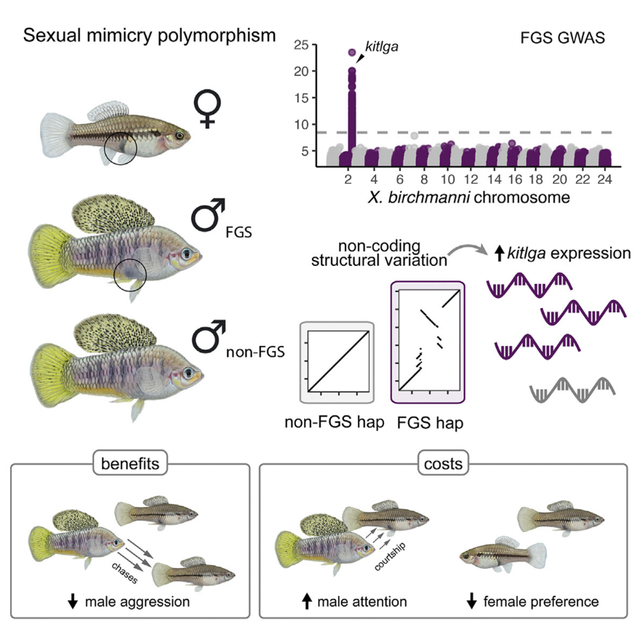

## INTRODUCTION

How diverse phenotypes arise and persist in a polymorphic state within interbreeding populations are longstanding questions in evolutionary biology.^1,2^ Over short timescales, such phenotypic polymorphisms could be caused by neutral alleles that have drifted to intermediate frequency, adaptive alleles moving toward fixation by directional selection, or demographic processes such as migration. However, balancing selection is the only mechanism known to maintain variation within populations over long timescales or at stable frequencies.^3,4^ Despite recent progress linking genetic and phenotypic variation to selection in nature,^5–8^ many classes of balanced polymorphisms remain incompletely understood. For instance, balancing selection is often invoked to explain the persistence of sexual mimicry polymorphisms—where males mimic females or vice versa.^9^ However, these phenotypes are generally poorly characterized at the genetic level (but see Kunte et al.,^10^ Andrade et al.,^11^ Sandkam et al.,^12^ and Willink et al.^13^). Sexual mimicry traits can be morphological,^14^ chemical,^15,16^ or behavioral,^17^ and may confer diverse benefits beyond alternative reproductive strategies,^18^ including harassment avoidance^14^ and physiological adaptation.^19^ Thus, sexual mimicry polymorphisms present exciting opportunities to characterize the genetic, evolutionary, and population-level processes that contribute to the maintenance of genetic and phenotypic variation in natural populations.

Theory predicts that discrete phenotypes segregating within populations should have a simple genetic basis.^20–22^ Concordantly, empirical work has uncovered many instances where single loci underpin phenotypic polymorphisms, although this may sometimes be driven by low power and discovery biases.^23,24^ However, the genetic basis of these polymorphisms can be diverse, ranging from single nucleotide changes^25^ to megabase-scale structural variation.^26–28^ Large inversions in particular, which limit recombination between coadapted alleles and allow suites of traits to be inherited as simple Mendelian loci,^29,30^ are frequently discovered in association with phenotypic polymorphisms.^26,31–35^ In contrast, the contribution of smaller structural changes—which play important roles in adaptation^36^ and disease^37,38^—to balanced polymorphisms are not well understood. Smaller structural variants have been difficult to identify and study due to limitations of short-read sequencing, but recent advances in long-read technologies make it possible to precisely interrogate these variants and link them to variation in phenotype.^39,40^

**Figure 1.**
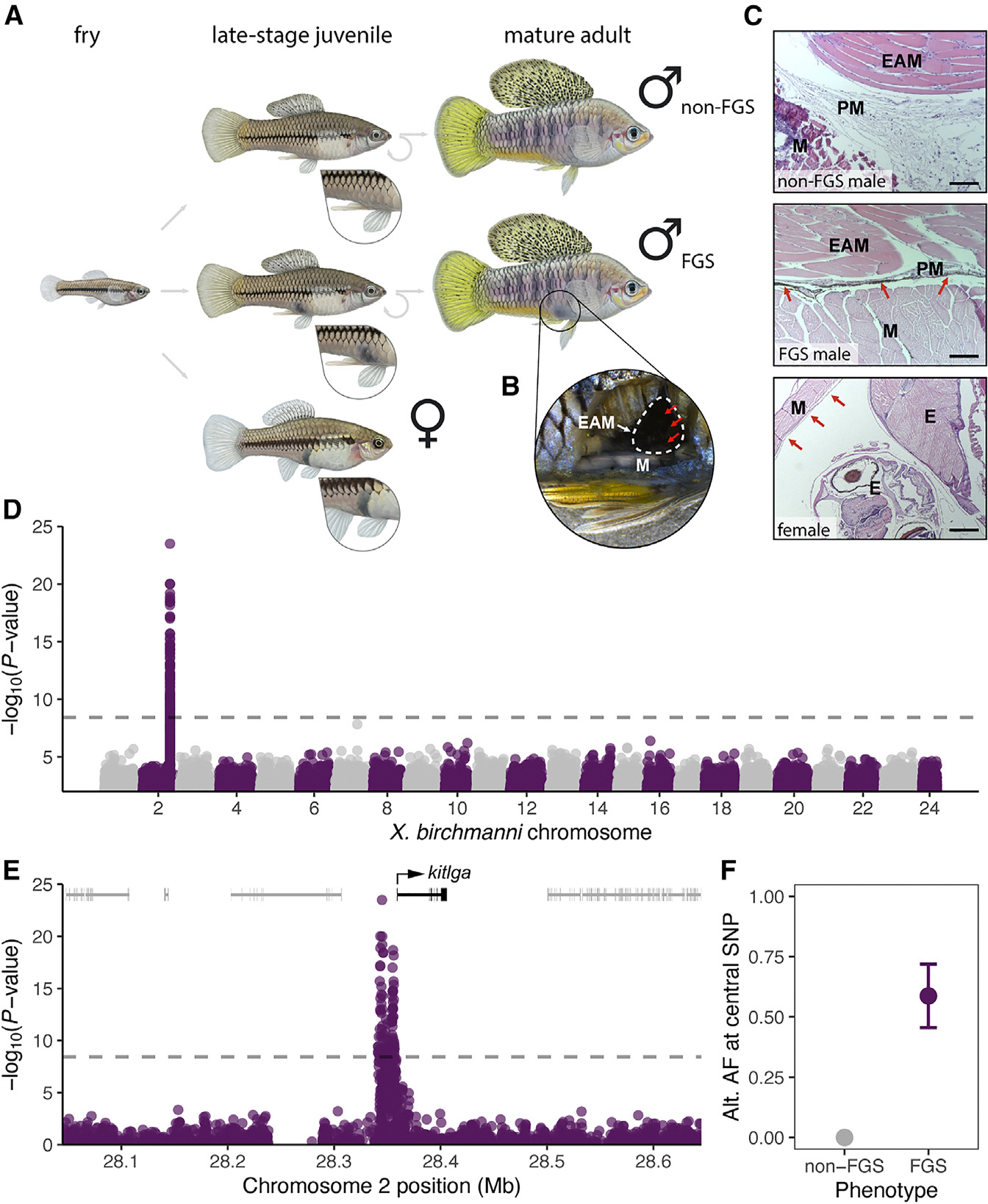
The false gravid spot is a pigmentation polymorphism in *X. birchmanni* with a simple genetic basis (A) Fry (left) are morphologically indistinguishable until the onset of puberty. Females (bottom) develop a gravid spot, while some males develop a false gravid spot (FGS, middle) in this region or develop no pigmentation (non-FGS, top). The FGS remains visible throughout the adult male’s life after sexual maturity. Illustrations by Dorian Noel. (B) FGS derives from pigmentation of internal tissues surrounding the gonopodial suspensorium, which is visible through the body wall musculature and skin. (C) Histological sections reveal that *X. birchmanni* males without FGS (top) do not develop pigmentation, while males with the spot accumulate melanophores in the perimysium of the erector analis major. The gravid spot is due to expansion of the pigmented peritoneum (bottom). Labels: M, body wall musculature; EAM, erector analis major; PM, perimysium of EAM; E, embryo; red arrows, pigmented melanophore cells. Scale bars denote 50 μm for top and middle images and 200 μm for bottom image. See also Figure S1. (D) Manhattan plot shows a single autosomal region on chromosome 2 that is associated with the FGS. Dashed line shows the 5% false positive threshold. See also Figure S2. (E) Inset highlighting 17 kb genome-wide significant region, occurring 1.5 kb upstream of the *kitlga* gene. See also Figures S3 and S4. (F) Allele frequency (AF) of the non-reference allele at the central representative SNP from the GWAS region (position 28,349,032) in non-FGS and FGS males. Error bars denote ±2 binomial standard errors.

Swordtail fish in the genus *Xiphophorus* are classic models of sexual selection driving the evolution of ornamentation^41–44^ and are an excellent system for studying the genetic architecture and selective forces underpinning polymorphic traits, including sexual mimicry phenotypes. Many *Xiphophorus* species are sexually dimorphic, with males displaying elaborated fin morphology and coloration.^43,45,46^ Additionally, females develop a gravid, or “pregnancy,” spot at sexual maturity, which becomes more pronounced during internal development of mature eggs and embryos.^47^ Enlarged gravid spots increase male courtship incidence in some *Xiphophorus* species^48^ and are hypothesized to have evolved to signal sexual receptivity.^47^ In several *Xiphophorus* species, some males develop a superficially similar melanic pigmentation trait, called the “false gravid spot” (Figure 1A). This phenotypic polymorphism was first described nearly a century ago^49^ and has been presumed to function in sexual mimicry.^50^ While polymorphisms shared by related species are often a sign of balancing selection,^51,52^ little is known about the selective forces acting on the false gravid spot, with only one study describing behavioral factors favoring the trait.^53^ Moreover, the genetic basis and developmental progression of this sexual mimicry polymorphism remain unknown.

Here, we find that variation in the false gravid spot phenotype in *X. birchmanni* is controlled by a non-coding region upstream of *kit ligand a* (*kitlga*), a gene known to underly pigment pattern variation in other species. The haplotypes associated with the false gravid spot display considerable structural complexity and drive higher allele-specific expression of *kitlga*. We demonstrate that the false gravid spot emerges in a male-specific tissue and develops before most other secondary sexual traits. Several lines of phenotypic and genetic evidence suggest that this polymorphism is maintained by balancing selection. In laboratory trials and observations in the wild, we find the false gravid spot influences behavioral interactions including aggression and mate choice, hinting that social interactions may contribute to the maintenance of this sexual mimicry polymorphism in nature.

## RESULTS

### The false gravid spot occurs in a male-specific tissue structure and is distinct from the female gravid spot

The gravid spot in female *Xiphophorus* develops during sexual maturation when the heavily melanized peritoneal lining surrounding the internal organs expands during oogenesis and becomes visible through the body wall.^47^ While the false gravid spot in males also arises during puberty,^49^ it develops slightly posterior to the position of the gravid spot in females and instead localizes above the gonopodium, the modified anal fin used for internal fertilization by male poeciliid fishes (Figure 1A). We therefore hypothesized that the anatomical basis of the false gravid spot and true gravid spot are distinct. Dissections in *X. birchmanni* males showed that pigmentation originates from the tissue surrounding the gonopodial suspensorium bone structure (Figures 1B and S1). Histological sections revealed melanophore accumulation in a thin perimysium surrounding the erector analis major muscle of males with the false gravid spot, while this perimysium was unpigmented in males lacking the trait (Figure 1C). The body wall and erector analis major musculature were unpigmented in all males, suggesting that the melanized perimysium drives the externally visible phenotype. Females lack an equivalent tissue structure, and melanophores instead localize to the peritoneal lining (Figures 1C and S1).

### Variation in the false gravid spot phenotype maps upstream of *kitlga*

To identify genomic regions controlling variation in the false gravid spot polymorphism in *X. birchmanni*, we performed a case-control genome-wide association study (GWAS) to search for allele-frequency differences between phenotype classes. Our sample consisted of 329 males from a single population with 39% false gravid spot frequency (Río Coacuilco), previously sequenced at low-coverage genome-wide.^41^ Using a newly assembled, highly contiguous and complete *X. birchmanni* reference genome from an individual without the false gravid spot (Table S1), our GWAS recovered a single peak on chromosome 2 (Figure 1D), which surpassed the genome-wide significance threshold estimated using simulations (*p* < 3.7 × 10^−9^). This association remained robust after correcting for population structure (*p* < 1 × 10^−10^; Figure S2). Chromosome 2 is expected to be an autosome in *X. birchmanni*, based on sex chromosome architecture in related species.^41,54^

The 17 kb region associated with the false gravid spot did not include any genes but was adjacent to *kitlga* (Figure 1E), a gene with a well-documented and conserved role in melanophore development and homeostasis.^55,56^ We found the proximal end of the associated region fell 1.5 kb upstream of the likely *kitlga* transcriptional start site (Figures 1E and S3). Within the significant region, we detected strong allele-frequency differences between phenotype groups (Figure 1F), consistent with a dominant and highly penetrant architecture of the false gravid spot. Analysis of the amino acid sequences of *kitlga* across multiple *X. birchmanni* individuals sequenced at high coverage revealed no variation (Figure S4), confirming the trait is not driven by coding differences. Moreover, comparisons to *X. birchmanni*’s sister species, *X. malinche*, indicated that rates of protein evolution in *kitlga* were consistent with strong purifying selection (dN/dS = 0.001; Figure S4).

### Complex structural variation is associated with the false gravid spot

**Figure 2.**
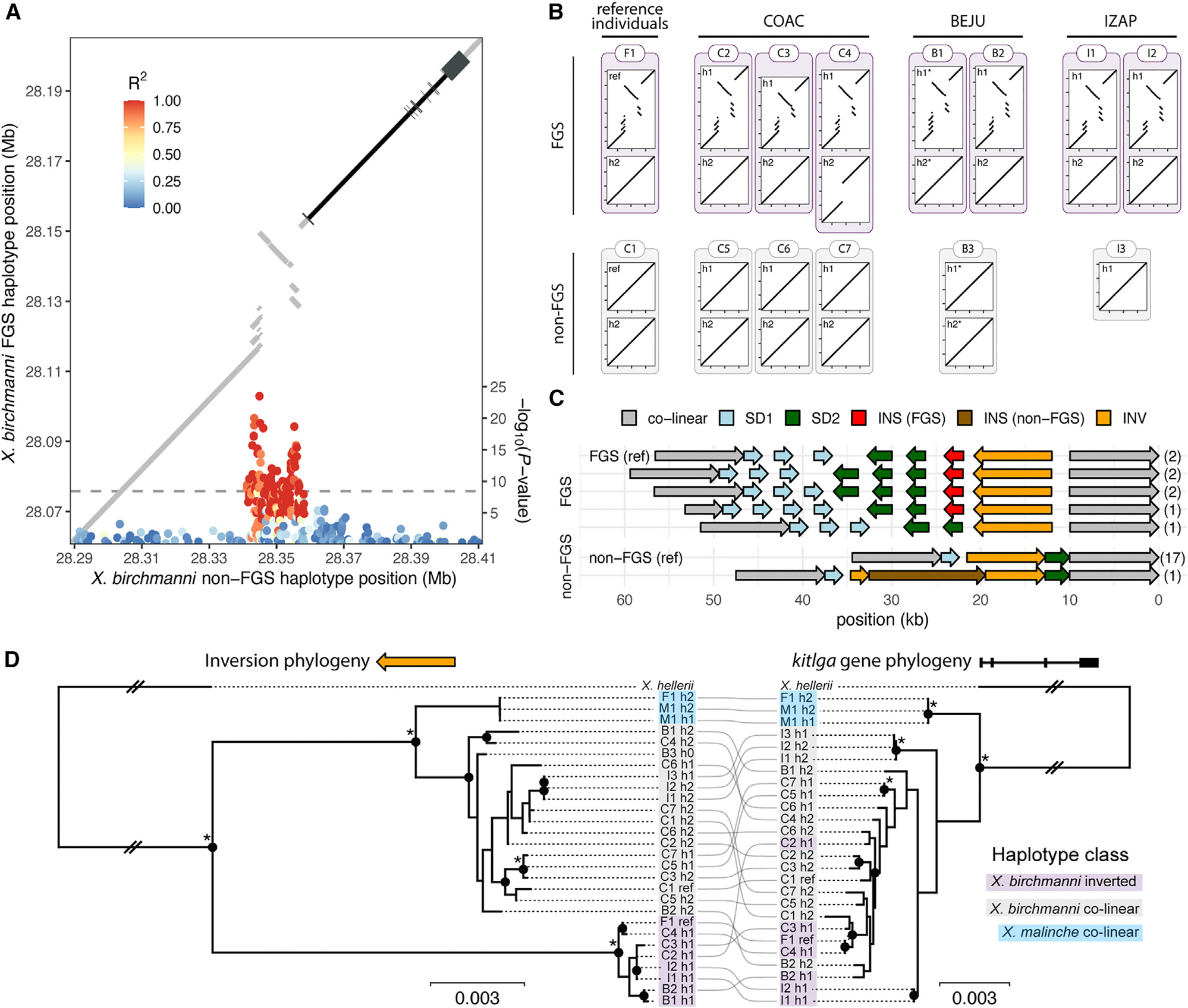
A complex structural variant is associated with the false gravid spot locus (A) Combined plot showing GWAS peak overlayed with MUMmer4 alignment (gray lines) of false gravid (FGS) and non-false gravid (non-FGS) haplotypes. Color of points in GWAS denotes R^2^ with the center SNP (position 28,349,032). Region of high LD colocalizes with the complex structural variant. The *kitlga* gene is plotted to the upper-right of the structural variant, with exons noted with thicker segments. See also Figures S5 and S6 and Table S2. (B) Long-read sequencing reveals that this complex structural variant is perfectly associated with the FGS phenotype across populations. Alignments between each haplotype compared with the reference non-FGS sequence. Individuals with the FGS phenotype are indicated in purple outlines, and individuals without are noted in gray. Reference individuals include the F_1_ *X. birchmanni* × *X. malinche* hybrid with FGS and the chromosome-level assembly from Coacuilco without FGS. An additional 12 individuals were sequenced across three *X. birchmanni* populations (COAC, Coacuilco; BEJU, Benito Juarez; IZAP, Izapa). Haplotypes were extracted from diploid assemblies, unless noted with a star, in which case individual long reads spanning the region were used to infer haplotype structure. The non-FGS individual from Izapa (I3) was completely homozygous in this region, so a single haplotype is displayed. (C) Five structurally variable haplotype classes exist across eight FGS haplotypes sequenced, compared with two classes across 17 non-FGS haplotypes in *X. birchmanni*. Numbers on right denote number of times haplotype was observed. SD1, segmental duplication 1; SD2, segmental duplication 2; INS (FGS), insertion in FGS haplotype (piggyback 4 element); INS (non-FGS), insertion in non-FGS haplotype; INV, inversion. See also Figure S7 to connect haplotype labels to structural variant classes. (D) Local phylogeny of the inversion (left) versus *kitlga* gene sequence (exons and introns; right) in *X. birchmanni* and *X. malinche*, with *X. hellerii* as the outgroup. Sequence names same as (B) with lines connecting the same haplotype. The inverted region clusters by structural variant type and then by species, while the genic sequence clusters by species and not by structural variant. Nodes with over 80% bootstrap support are noted with black circles, and nodes with 100% support are labeled with an additional asterisk. Slashes indicate where branch lengths were shortened for visualization. See also Figure S8.

An initial investigation into haplotype structure of the associated region using higher coverage short-read data (median 18.4×, 23 individuals) revealed strong linkage disequilibrium (LD) within the GWAS peak and a rapid decay on either side (Figure 2A). To explore if this LD pattern was driven by structural differences between alternate alleles, we turned to long-read data due to limitations of short-read methods for identifying structural variation.^57^ We produced a PacBio HiFi assembly for the false gravid spot haplotype and generated a pairwise alignment between this sequence and the non-false gravid spot haplotype with MUMmer4.^58^ We found evidence of complex rearrangements between alternate alleles, which precisely localized with the strongest signal in the GWAS upstream of *kitlga* and the region of high LD (Figure 2A).

We next tested if the structural variant was consistently associated with the false gravid spot by conducting long-read sequencing across the *X. birchmanni* species range. We generated *de novo* diploid assemblies for 12 additional individuals from three populations using Oxford Nanopore Technologies (ONT) or PacBio HiFi long-read sequencing. In all *X. birchmanni* individuals with the false gravid spot phenotype (*n* = 7), we identified one rearranged haplotype and one haplotype that was largely co-linear with the non-false gravid spot reference (Figure 2B). In individuals without the false gravid spot (*n* = 6) all haplotypes were co-linear with respect to the reference (Figure 2B). Together, these results strongly connect the presence of the rearranged haplotype with the false gravid spot phenotype (*p* = 0.0013, Fisher’s exact test) and indicate that this haplotype is dominant with respect to phenotype. While all wild-caught *X. birchmanni* with false gravid spot in our long-read dataset were heterozygous for the rearrangement, this is not unexpected given the sample size (*p* = 0.3976 by simulation), and we detected two individuals homozygous for SNPs diagnostic of the false gravid spot allele in our larger high-coverage short-read dataset.

In pairwise alignments between the two reference haplotypes (Figure 2A), the complex structural variant contained two distinct segmental duplications, an insertion, and an inversion (Figure S5). Within the rearrangement, the two distinct duplications and the inversion were alignable between the false gravid and non-false gravid haplotypes, although divergence between the haplotypes was high (Figure S5; 93%–97% identity versus >99.5% outside of the rearrangement). In contrast, 2 kb of the insertion showed high similarity to a *Xiphophorus* piggybac protein 4 transposable element (Table S2). The false gravid spot haplotype also had higher frequency and length of non-canonical DNA structures, namely inverted repeats (Figure S6). To identify additional structural differences across haplotypes, we extracted each structurally distinct component of the pairwise MUMmer4 alignment (e.g., duplications, inversion, insertion) and mapped them to each phased diploid assembly to obtain regions of homology. This approach revealed five distinct structural architectures across eight false gravid spot haplotypes sequenced (Figures 2C and S7). Differences among false gravid spot alleles include copy-number variation in the distal segmental duplication (SD1; 3 versus 4 copies), proximal segmental duplication (SD2; 2 versus 3 copies), presence of the insertion (0 versus 1 copy), and length of the region between the two segmental duplications (1 versus 4 kb). In contrast, we detected only two structural architectures among the 18 non-false gravid spot haplotypes sequenced: 17 haplotypes were structurally identical to the reference, and one haplotype had a 20 kb insertion.

To better understand the evolutionary history of the false gravid spot locus, we constructed a local phylogeny of the structurally variable region and compared it to that of the *kitlga* gene. We included all long-read assemblies from *X. birchmanni* as well as long-read data from its sister species *X. malinche*, which lacks the false gravid spot. As an outgroup, we used the long-read *X. hellerii* assembly,^59^ a distantly related *Xiphophorus* species without the false gravid spot. The assemblies from both *X. malinche* and *X. hellerii* were co-linear with the non-false gravid allele in *X. birchmanni* (Figure S8). For the structural variant tree, we focused on the 8.8 kb inversion, which was present as a single copy across all haplotypes. We extracted this region and the *kitlga* gene sequence (including introns) from each assembly and constructed local maximum likelihood phylogenies with RAxML^60^ (Figure 2D). For the inversion, we found the false gravid spot and non-false gravid spot haplotypes formed distinct, monophyletic groups. Within the non-false gravid spot haplotypes, *X. birchmanni* and *X. malinche* grouped separately. Taken together, this suggests the false gravid spot and non-false gravid spot *X. birchmanni* haplotypes diverged prior to divergence of *X. malinche* and *X. birchmanni*. However, the same was not true for the *kitlga* gene, which clustered by species but not by phenotype. Together, this phylogenetic analysis highlights a history of recombination between the structural variant and the *kitlga* gene and underscores the importance of the structural variant in producing the false gravid spot.

### Increased tissue-specific expression of *kitlga* in false gravid spot males

**Figure 3.**
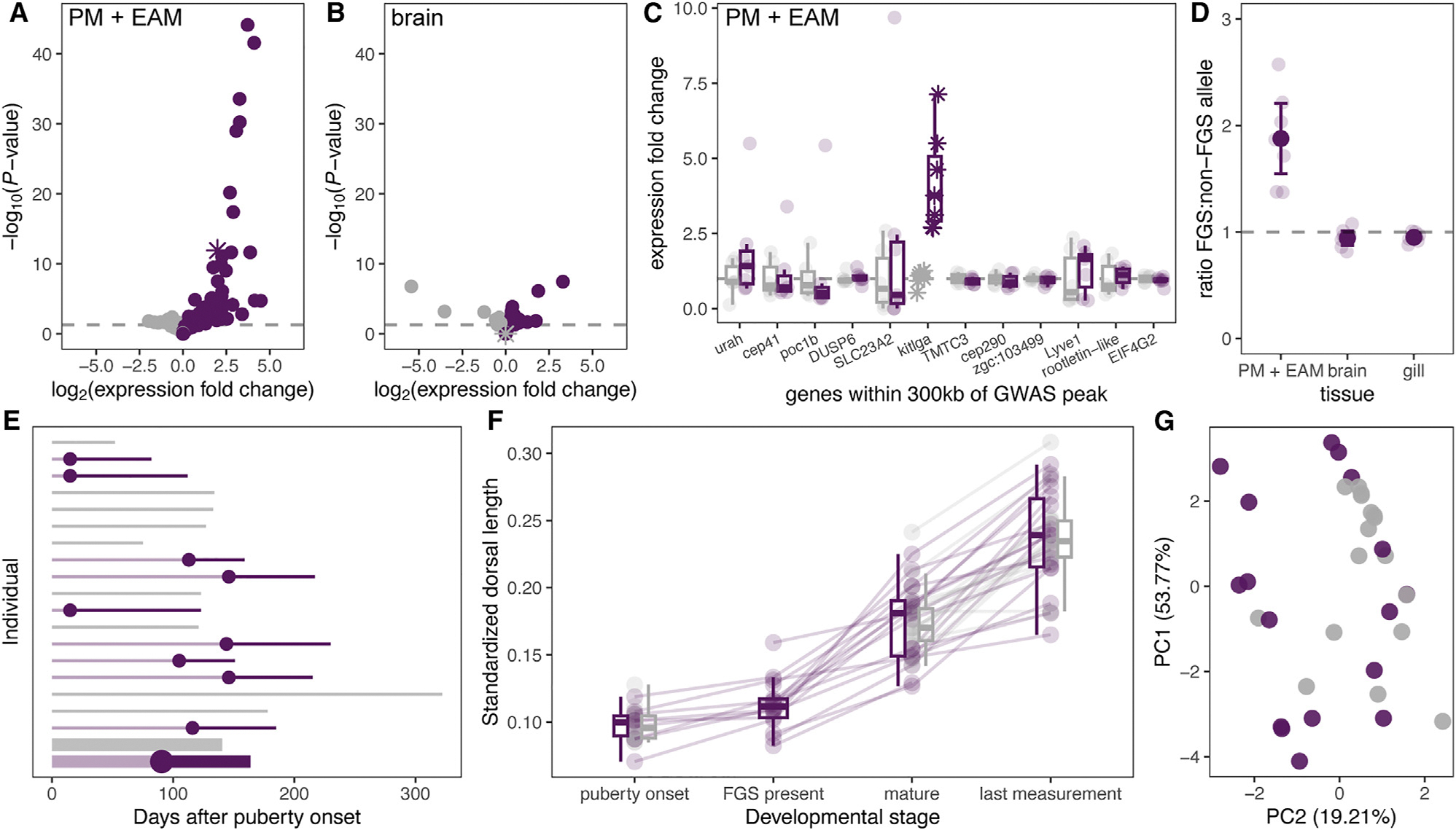
Gene expression and developmental timing of the false gravid spot phenotype (A) RNA-seq analysis shows differential gene expression in individuals with and without false gravid spot (FGS) in the erector analis major tissue and its perimysium (EAM + PM). *kitlga* is shown with a star. Genes with increased expression in FGS individuals are shown in purple, and genes with increased expression in non-FGS individuals are shown in gray. See also Table S3. (B) Brain tissue shows limited differential gene expression between FGS and non-FGS individuals, including for *kitlga*. See also Table S3. (C) In EAM + PM tissue, *kitlga* is the only differentially expressed gene within 300 kb of the GWAS peak. Expression fold change of the 12 closest genes to the peak (ordered by genomic position), normalized by mean non-FGS individual expression, with data for non-FGS individuals in gray and FGS individuals. (D) Allele-specific expression in *X. birchmanni* × *X. malinche* hybrids with FGS suggests the expression differences in *kitlga* are under *cis-*regulatory control. Expression is normalized to the *X. malinche* (non-FGS) allele. Large points and whiskers denote mean ± 2 binomial standard errors, and small points represent individuals. See also Figure S9. (E) Developmental time between onset of puberty until sexual maturity in 17 *X. birchmanni* males in the laboratory. Individuals are ordered from top to bottom by birth date; FGS individuals are noted in purple, with the dot showing when the spot developed; and non-FGS individuals are in gray. Thick bars and dot at the bottom represent mean time to sexual maturity and FGS development timing for each group. See also Figure S10 and Table S5. (F) Dorsal fin length, standardized by body length, across fish from the developmental series shows that dorsal fin elongation tends to occur later during sexual maturity and continues to elongate after males are reproductively mature. (G) Principal-component analysis (PCA) of male phenotypes at sexual maturity shows FGS and non-FGS males raised in laboratory conditions do not systematically differ in their secondary sexual characteristics (morphometrics and pigmentation traits).

Given that the structural rearrangement localizes to a non-coding region and there is no evidence of *kitlga* amino acid variation, we investigated if expression differences in *kitlga* might drive the false gravid spot phenotype. We performed mRNA sequencing in 13 juvenile males with and without false gravid spot and analyzed the data using pseudoalignment with kallisto^61^ and differential expression analysis with DESeq2^62^ (Table S3). *kitlga* was among the most differentially expressed genes genome-wide between false gravid spot and non-false gravid spot males in the erector analis major and its perimysium (Figure 3A) but was not differentially expressed in the brain (Figure 3B). We also found increased expression of several other genes with known roles in melanogenesis—including *pmel*, *tyrp1*, *mlana*, and *oca2* (Table S3)—consistent with increased melanophore abundance in the perimysium of the erector analis major in males with false gravid spot. However, *kitlga* was the only gene within 300 kb of the GWAS peak that was differentially expressed in this tissue (Figure 3C), suggesting it might act upstream of other differentially expressed genes.

The position of the structural variant near the predicted *kitlga* transcriptional start site suggests that it could modulate expression as a *cis*-regulatory element, a hypothesis we tested by measuring allele-specific expression. Given that few SNPs segregate in the *X. birchmanni kitlga* coding sequence, we measured allele-specific expression in laboratory-generated *X. birchmanni* × *X. malinche* hybrids with the false gravid spot. While the non-false gravid spot haplotypes in *X. malinche* and *X. birchmanni* are structurally similar (Figure S8), the *kitlga* coding sequence has accumulated several synonymous substitutions between species (Figure S9), which allow us to distinguish whether a transcript originated from the false gravid spot (*X. birchmanni*) or non-false gravid spot (*X. malinche*) haplotype. Using a pyrosequencing approach (Table S4), we found strong differential expression of the false gravid spot and non-false gravid spot alleles in the erector analis major and perimysium (Figures 3D and S9; *p* < 0.001) and little evidence of differential expression in other tissues (Figure 3D). Together, this suggests that the structural variant drives increased allele-specific and tissue-specific expression of *kitlga*.

### The false gravid spot develops before sexual maturity and precedes other secondary sexual traits

To determine when the false gravid spot develops, we tracked the phenotypic progression of 43 males raised together in standard conditions from the onset of puberty through >1 year of age. We defined the onset of puberty as when males began to develop a gonopodium and defined completion of sexual maturity as when the gonopodium became a functional intromittent mating organ^63–65^ (Figure S10; Table S5). We found that males begin to develop the false gravid spot area coincidently with the early stages of external gonopodium development, in many cases several months before they completed sexual maturity (Figure 3E). This is expected given that modification of anal fin rays that form the gonopodium coincides with the development of the gonopodial suspensorium, to which the erector analis major and its perimysium are anchored.^66^

In addition to preceding sexual maturity, the false gravid spot also developed before two important sexually dimorphic traits in *X. birchmanni*, the dorsal fin and vertical bars, which are used for intra- and inter-sexual communication in *Xiphophorus*.^67,68^ Most elongation of the dorsal fin occurred after false gravid spot development (Figure 3F). However, lab-reared males with and without the false gravid spot did not significantly differ in overall ornamentation in adulthood (Figure 3G). This suggests that while the false gravid spot develops before sexual maturity, it is not associated with variation in other sexually dimorphic ornaments in adult fish raised under laboratory conditions.

### The false gravid spot shows evidence of being a balanced polymorphism

**Figure 4.**
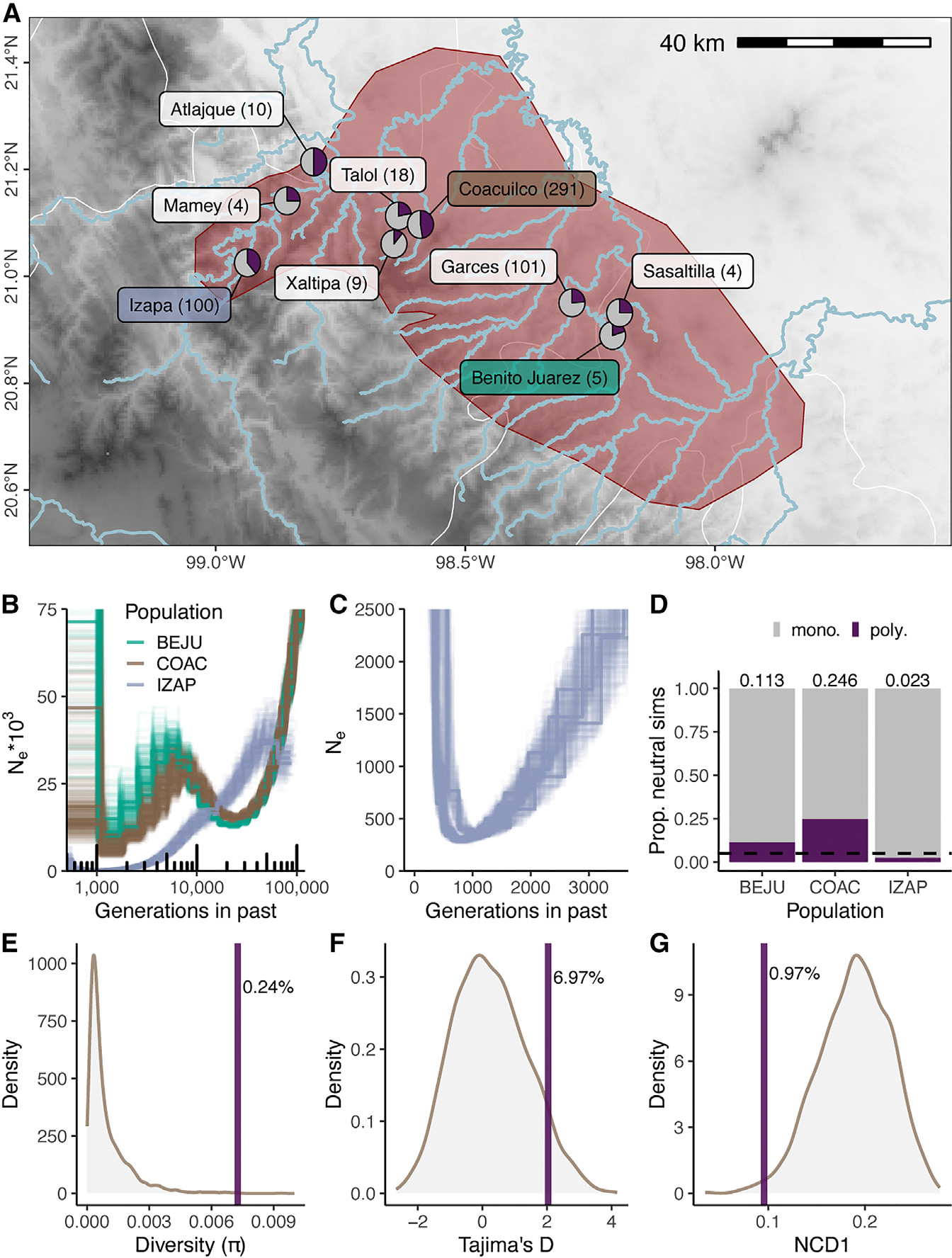
The false gravid spot is under balancing selection in the wild (A) The false gravid spot (FGS) is present in all sampled populations across the *X. birchmanni* range (red polygon). Pie charts depict FGS phenotypic frequency in adult males, ranging from 0.11 to 0.50, with sample size in parentheses. Map shows elevation from 0 (white) to 3,000 m (black), with major rivers shown in blue and state boundaries shown in white. See also Figure S11. (B) PSMC plot showing inferred population histories over the last ~100,000 generations of three *X. birchmanni* populations, Benito Juarez (BEJU), Coacuilco (COAC), and Izapa (IZAP), with variants called from ONT data. Izapa experienced a distinct demographic history from Benito Juarez and Coacuilco, including a sustained bottleneck. (C) Recent population history at Izapa inferred from PSMC, showing a minimum N_e_ of 290 individuals around ~1,000 generations before the present. (D) Outcome of simulations of neutral polymorphisms under the demographic history inferred for each population from PSMC (simulating 63,000–73,000 generations). In the absence of selection, polymorphisms are maintained in only 2.3% of simulations (*n* = 10,000) matching the demographic history in Izapa. (E) Nucleotide diversity (π) within the inverted region in 23 individuals from Coacuilco compared with the distribution of all 8.8 kb windows from chromosome 2. The π value calculated in the inversion is in the top 0.24% chromosome-wide. (F) Tajima’s D within the inversion falls within the top 6.97% of values chromosome-wide. (G) The NCD1 value within the inversion is in the bottom 0.97% of values chromosome-wide.

With a better understanding of the genetic and developmental basis of the false gravid spot, we investigated if selection may be acting on the trait in nature. Based on expectations under some models of balancing selection, we predicted the false gravid spot would exhibit similar frequencies across populations and not be fixed at any location. Across nine *X. birchmanni* populations (542 total individuals) spanning the species range, we found the false gravid spot was polymorphic in all locations. In all populations, phenotypic frequencies ranged from 0.11 to 0.50 (Figure 4A) and were between 0.24 and 0.47 at three geographically distinct sites with higher confidence frequency estimates (≥100 individuals sampled). At Coacuilco, a site we repeatedly sampled from 2017 to 2023, we found the phenotypic frequency fluctuated between 0.30 and 0.60 during this interval (Figure S11), with some significant changes between years (two proportion Z test, *p* = 0.015).

Since some of the *X. birchmanni* populations surveyed occur in different river drainages, we next explored if the demographic history of these populations might inform our understanding of false gravid spot maintenance. Leveraging the long-read data we collected from Coacuilco, Izapa, and Benito Juarez, we ran PSMC,^69^ which implements the pairwise sequential Markovian coalescent to infer demographic histories (Figure 4B). We discovered that the Izapa population underwent a sustained bottleneck (estimated N_e_ as low as 293 individuals) for several thousand generations but has a contemporary false gravid spot frequency of 39% (Figure 4C). To explore the likelihood of false gravid spot maintenance in the absence of selection, we ran SLiM^70^ simulations of a neutral locus in populations matching the inferred demographic histories. We found a neutral locus was maintained as polymorphic in only 2.3% of all neutral simulations matching the demographic history of Izapa (Figure 4D; *n* = 10,000 simulations). This suggests that selection likely plays a role in maintenance of the false gravid spot in at least some *X. birchmanni* populations.

Finally, we investigated if the false gravid spot locus and linked regions displayed patterns of genetic variation consistent with balancing selection.^71,72^ We again leveraged the high coverage short-read dataset from the Coacuilco population, which had been collected agnostic to phenotype. Because we anticipated challenges calling variants from short-read data near the copy-number variable regions within the locus, we focused on the 8.8 kb chromosomal inversion. Compared with windows of the same size across chromosome 2, we found patterns of variation within the inversion consistent with balancing selection. The inversion displayed elevated nucleotide diversity (π; top 0.24%; Figure 4E) and Tajima’s D (top 6.97%; Figure 4F). Finally, the within-population non-central deviation statistic (NCD1), which has improved power compared to Tajima’s D,^72,73^ was also an outlier (bottom 0.97%; Figure 4G). We repeated this analysis considering only 8.8 kb windows that fell in the lower 8% quantile of recombination rate, matching the inferred recombination rate in the inverted region, and found similar results (π, top 0.33%; Tajima’s D, top 3.15%; NCD1, bottom 1.89%).

### Behavioral interactions provide clues about the mechanisms of selection

**Figure 5.**
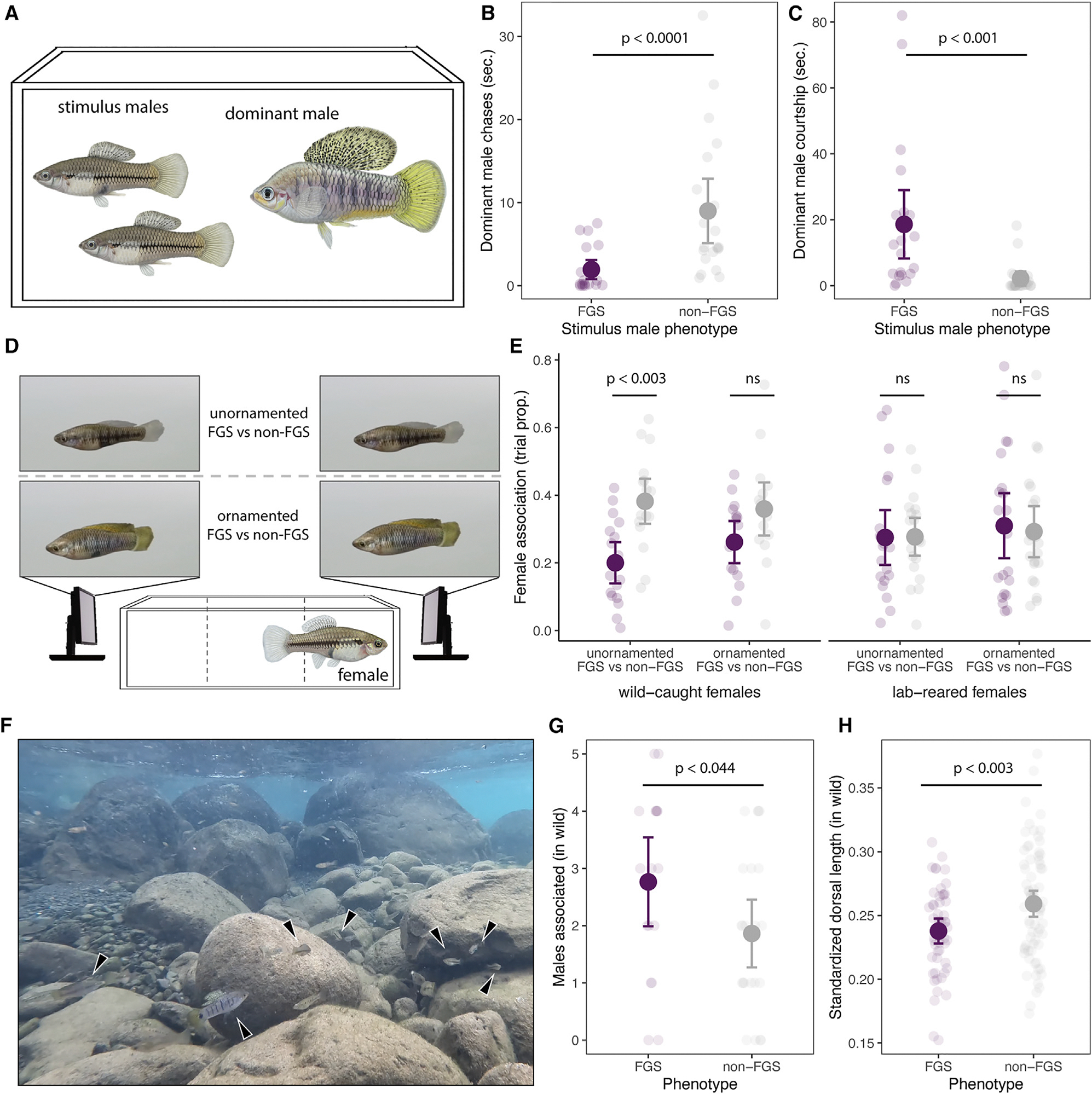
Behavioral consequences of the false gravid spot (A) Diagram of male-male interaction experimental design. Scored interactions were all initiated by the focal large-bodied, dominant male directed toward two smaller, unornamented males, with and without false gravid spot (FGS). (B) FGS males were chased for less time compared to non-FGS males (paired Wilcoxon test; *p* < 0.0001). Large points represent experiment means ±2 standard errors, with small points denoting mean value for each focal male. (C) In the same trials, FGS males were courted more than non-FGS males (paired Wilcoxon test; *p* < 0.001). (D) Diagram of female preference experimental design. Females were presented a choice between FGS and non-FGS males in two animation types, unornamented males and ornamented males. (E) Females collected in the wild spend a greater proportion of the trial with animations of unornamented males without the FGS (paired Wilcoxon test; *p* < 0.003), suggesting disdain for the FGS. A significant difference in preference was not detected in any other stimulus or in a cohort of females born in the lab. See also Figure S12. (F) Still image from a representative site during observations in the Río Coacuilco, with *X. birchmanni* noted with arrows. (G) In the wild, FGS males had more males in their immediate vicinity (1 m^2^; GLM likelihood ratio χ^2^_1_ = 4.1, *p* < 0.044). See Figure S13 for how the FGS and other traits mediate additional behavioral interactions. (H) Wild *X. birchmanni* males with FGS have shorter dorsal fins than non-FGS males (Welch’s t test; *p* < 0.003). Dorsal length is represented as a fraction of body length. See also Figure S14.

Since multiple lines of evidence suggest the false gravid spot is under balancing selection, we next investigated mechanisms that may favor or disfavor this polymorphism. Because male *Xiphophorus* aggressively guard access to food resources and mates,^74–76^ we hypothesized that false gravid spot males may experience less aggression. We designed experimental triads in the lab consisting of one large dominant male and two size-matched smaller males, one with and one without the false gravid spot (Figure 5A). Because the false gravid spot precedes development of other sexually dimorphic traits, we chose smaller males that lacked ornamentation and thus may be more convincing female mimics at this life stage. Strikingly, all dominant males (*n* = 21) chased males with false gravid spots less than the paired stimulus male without the false gravid spot (Figure 5B; *p* < 0.0001). Whether or not the dominant focal male had false gravid spot did not significantly affect the frequency of aggressive interactions initiated (Welch’s t test; *p* < 0.086). Moreover, we also did not detect differences in boldness between phenotypes of males raised in the laboratory using a scototaxis assay (Welch’s t test; *p* < 0.78).

While experiencing reduced aggression would likely benefit false gravid spot males, being perceived as female may lead to a cost if females experience greater harassment.^14,77–79^ Because *X. birchmanni* court potential mates vigorously and males in other *Xiphophorus* species are known to attend to intensity of gravid spots when directing courtship toward females,^48^ we also hypothesized males with the false gravid spot may receive additional sexual attention from the dominant male. Consistent with this, in our experimental triads, we observed that dominant males spent more time courting false gravid spot males than they did non-false gravid spot males (Figure 5C; *p* < 0.001). Taken together, our laboratory behavioral assays demonstrate the false gravid spot is an important signal that influences interactions with other males.

Because female *Xiphophorus* choose mates based on a variety of traits,^45,67^ including pigmentation,^44,68,80^ we hypothesized the false gravid spot might be involved in female mate choice. However, the false gravid spot develops before male-diagnostic sexually dimorphic traits but remains expressed throughout the male’s life, and it was unclear if female preference might depend on the presence of other male-diagnostic traits. To assay preference while controlling for other phenotypes, we ran dichotomous choice tests using video animations of males with and without false gravid spot (Figure 5D). We showed females two animation types: unornamented males (i.e., small dorsal fin and muted coloration) and ornamented males (i.e., large dorsal fin and strong coloration). Moreover, because female mate preference in swordtails often depends on experience,^81–83^ we simultaneously tested a cohort of wild-caught females and a cohort reared in the laboratory with no previous mate-choice experience. Females from the wild associated 40% less with false gravid spot males in the unornamented phenotypic background (Figure 5E; *p* = 0.003), suggesting females disdain the false gravid spot in some contexts. We also found a non-significant trend for disdain of the false gravid spot in the ornamented male animations (Figure 5E; *p* = 0.11). By contrast, we did not find evidence of preference for or against the false gravid spot in females that were born in the laboratory (Figure 5E; *p* = 0.89). Simulations revealed good power to detect large effect sizes in our experiments using wild-caught females (e.g., ~90% power for the effect in the unornamented male experiment) but low power to detect weaker effect sizes (e.g., 32% for the effect in the ornamented male experiment). This suggests that we cannot rule out the possibility that female disdain for the false gravid spot is merely weaker for ornamented males (Figure S12).

Based on insights from these laboratory experiments, we next evaluated impacts of the false gravid spot in its natural context. During behavioral observations of 51 individuals conducted in the Río Coacuilco (Figure 5F), we found that inter- and intra-sexual social interactions were generally less frequent than in the laboratory experiment, and both male and female *X. birchmanni* spent most of their time swimming against the current or feeding. However, males with the false gravid spot were associated with significantly more males in close proximity (Figure 5G; generalized linear model [GLM] likelihood ratio χ^2^_1_ = 4.1, *p* < 0.044). Because the increased number of nearby males could be consistent with either the false gravid spot reducing male-male aggression or increasing male-male courtship (Figure S13), we assessed the condition of males with false gravid spot. In other *Xiphophorus* species, growth rate and size of secondary sexual ornaments depends on food availability and the amount of aggression males experience.^53,84^ While we did not detect differential growth rates of juveniles with and without false gravid spot (Figure S14), we found the dorsal fin was on average 10% smaller in *X. birchmanni* males with the false gravid spot in the wild (Figure 5H; Welch’s t test, *p* < 0.003).

## DISCUSSION

While often hypothesized to be under balancing selection, there are few examples of sexual mimicry polymorphisms where both the causal genes and mechanisms of selection are understood. Here, we identify the genetic and developmental basis of a female mimicry phenotype in the swordtail fish *X. birchmanni* and uncover behavioral mechanisms that may play a role in its maintenance. Previous studies linking genomic variation to sexual mimicry polymorphisms have uncovered genes in the sex determination pathway^10^ or regions within the non-recombining portions of the sex chromosome^12^ (but see Willink et al.^13^). In contrast, we find that the false gravid spot in *X. birchmanni* is controlled by a small structural variant upstream of the *kitlga* 5′ UTR. *Kitlga* (and *kitlgb*) arose from *kitlg*, a well-described pigmentation gene,^55^ during the ancient teleost whole-genome duplication. Prior work indicates that *kitlga* has maintained the ancestral pigmentation function in multiple teleost species^85,86^ and has been linked to pigmentation variation in natural populations of stickleback^56^ and in domesticated populations of betta fish.^87^ Thus, our findings suggest that a well-known teleost pigmentation gene underpins the false gravid spot sexual mimicry phenotype.

While the contribution of structural variation to the evolution of balanced polymorphisms is well appreciated, much of the focus has been on large chromosomal inversions, which may function as supergenes.^29–35^ Smaller structural variants, including those acting on single genes to generate phenotypic polymorphisms, have been previously described^8,88,89^ but these have largely been interrogated with short-read data and coverage-based analyses, approaches with more limited resolution and precision. Using long-read sequencing and *de novo* genome assembly, we fully resolve complex structural variation that drives the false gravid spot phenotype. The five structural architectures we identify among the eight false gravid spot haplotypes sampled stand in contrast to the simple non-false gravid spot sequence, where we detect only two structural architectures among 18 haplotypes (Figure 2C). The high structural diversity of the false gravid spot haplotypes lends support to the idea that structural variation can predispose sequences to the evolution of additional structural variation, driven by the complexities of DNA replication, repair, and recombination in such regions.^90,91^

The location of this structural variant near the *kitlga* transcriptional start site suggested that it may act by altering its expression. Together, our RNA sequencing (RNA-seq) and allele-specific expression results demonstrate that the localization of the structurally complex haplotype upstream of *kitlga* results in both tissue-specific and allele-specific upregulation of *kitlga* in males with false gravid spot. Prior work has suggested that *kitlg* and *kitlga* drive pigmentation phenotypes in natural populations largely through *cis-*regulatory changes.^92^ While precise genetic changes underlying *kitlg* regulation have been identified in humans, where a SNP within an enhancer impacts hair color,^93^ the causal variants affecting *kitlga* expression have remained elusive in fish. Although it remains unclear which component of the false gravid spot structural variant might drive variation in expression, copy-number variation in non-coding regions has previously been associated with expression quantatative trait loci (eQTL) and disease phenotypes.^90,94^ Interestingly, long-read sequencing in humans has revealed that smaller inversions (1–20 kb) are often associated with repeat expansions.^91^ Although the importance of these variants is largely unknown, our findings raise the possibility that smaller-scale structural variation may be an important driver of phenotypic polymorphisms by impacting gene regulation. Addressing this question is newly tractable in the era of long-read sequencing and chromatin accessibility assays.

Several lines of phenotypic and genetic evidence show that balancing selection is maintaining the false gravid spot polymorphism in *X. birchmanni*. To gain insight into specific selective mechanisms, we investigated behavioral interactions and found the false gravid spot affects multiple social interactions. One clear benefit of female mimicry is that males with the false gravid spot experience less aggression from other males, reducing risk of direct injury from such interactions, which can result in mortality in the lab environment. However, we also identified two potential costs of the false gravid spot. First, despite being chased less, males with false gravid spot were courted more by other males. While these interactions are not directly competitive, harassment may be energetically costly and time spent being courted represents time that males may not be able to feed.^14,78,79^ In the wild, we found that false gravid spot males also had smaller ornaments, which could be consistent with an energetic cost.^53^ Additionally, females disdain males with the false gravid spot in some situations, which could drive differential mating success via female mate choice. Taken together, the false gravid spot phenotype likely impacts fitness in natural populations.

While we see evidence for both costs and benefits of the false gravid spot, how exactly they interact to maintain the trait in natural populations remains unclear. Theory suggests that opposing fitness effects alone can only maintain balanced polymorphisms under a narrow range of circumstances.^95–97^ Our data may be consistent with several different forms of balancing selection where selection coefficients vary based on context, such as spatiotemporally varying selection and negative frequency-dependent selection. While we did not test for these mechanisms directly, our behavior experiments may hint at some context dependence. For instance, females displayed strong disdain for the false gravid spot in unornamented animations but showed weaker evidence of discrimination against false gravid spot males in ornamented animations (though we note that this may be impacted by power). Moreover, strength of preference may also vary as a function of female experience. We speculate that context dependence in social interactions could allow the false gravid spot to be maintained by frequency-dependent selection or spatiotemporally variable selection, which are commonly involved in alternative mating systems.^22,64^ Disentangling these possibilities and uncovering other selective mechanisms that impact this naturally occurring balanced polymorphism represent exciting directions for future work.

## RESOURCE AVAILABILITY

### Lead contact

Further information and requests for resources and reagents should be directed to and will be fulfilled by the lead contact, Molly Schumer (schumer@stanford.edu).

### Materials availability

This study did not generate new unique reagents.

### Data and code availability

- Sequencing data, including long-read whole-genome sequence (WGS) data, short-read WGS data, and RNA-seq data, have been deposited at NCBI sequence read archive (SRA) and are publicly available as of the date of publication. Accession numbers are listed in the key resources table. This paper also analyzes existing, publicly available sequencing data. These accession numbers for the datasets are listed in the key resources table. Image data and diploid assemblies have been deposited at Dryad and are publicly available as of the date of publication. DOIs are listed in the key resources table.
- All original code has been deposited on Github (https://github.com/tododge/xbirchmanni_fgs/ and https://github.com/Schumerlab/) and is publicly available as of the date of publication.
- Any additional information required to reanalyze the data reported in this paper is available from the lead contact upon request.

## STAR★METHODS

### EXPERIMENTAL MODEL AND STUDY PARTICIPANT DETAILS

*X. birchmanni* samples used for this study were collected using baited minnow traps from sites across the Mexican states of Hidalgo, San Luis Potosí, and Veracruz, with permission from the Mexican government and Stanford University animal welfare protocols (Stanford APLAC protocol #33071). Fish were housed at the Stanford fish facility in groups in 10–115 L tanks on a 12/12 light dark cycle at 22°C and fed twice daily. Juvenile and adult males from the genome-wide association study were collected from the Río Coacuilco (21°5’51.16”N 98°35’20.10”W) between 2017 and 2018 for a previous association mapping experiment.^41^ Adult males used to generate long-read assemblies were collected between 2021–2023 from Coacuilco, Benito Juarez (20°52’51.51”N 98°12’24.05”W), and Izapa (21°1’22.81”N 98°56’16.99”W). Juvenile males used in gene expression analyses were born and raised in the Stanford fish facility between 2021–2023. Fish tracked to characterize development were born in the Stanford fish facility in 2020 and followed to >1 year of age. Adult males and females tested in behavior experiments were collected from Coacuilco between 2020 and 2023, except when otherwise noted. Before behavioral experiments, fish were housed in single sex colonies for at least two weeks prior to testing. Juvenile males used to assess growth rates in the wild were collected in 2023 from Coacuilco. In most experiments that required lethal sampling, fish were euthanized with an overdose of MS-222 followed by severing of their spinal cord. However, in the gene expression experiments, fish were anesthetized on ice and euthanized by severing the spinal cord.

### METHOD DETAILS

#### Morphological analyses and histology

To examine the structure of the false gravid spot, male *X. birchmanni* were dissected under a microscope. Two non-false gravid spot males were dissected. For the gross dissections, three anatomical components of the false gravid spot were identified. We also examined the structure of the false gravid spot using a histology-based approach. We fixed two male *X. birchmanni* with the false gravid spot, two without, and two female *X. birchmanni* in a 4% formaldehyde solution for 24 hours at 4°C. Following fixation, the tissue was dehydrated, embedded in paraffin and we generated transverse sections for imaging at 5 μm thickness. These sections were stained with hematoxylin and counterstained with eosin.

#### Sample phenotyping

To obtain phenotypes, fish were anesthetized in a buffered solution of MS-222 at a concentration of 100 mg/mL and photographed with their fins spread on a grid background, using a Nikon d90 DSLR digital camera with a macro lens. Because the lighting environment was not standardized for photographs taken in the field, we recorded binary phenotypes for presence or absence of false gravid spot. We took quantitative measurements of male standard length, body depth, dorsal fin length and height using Fiji.^118^ We also recorded presence of melanic patterns (e.g., vertical bars, horizontal line, carbomaculatus, spotted caudal) and xantho-erythrophore coloration.

#### Genome wide association study

We reanalyzed previously published low-coverage (~0.35 ×) whole-genome Illumina data from 329 male *X. birchmanni* collected from the Coacuilco population between 2017 and 2018.^41^ The false gravid spot segregated at intermediate frequency in this natural population (126 with and 203 without). Because the false gravid spot occurred at similar frequencies in adult (0.39) and juvenile (0.38) males, we included both juveniles and adults in the association mapping analysis. We mapped all reads to the new *X. birchmanni* reference genome (see below) using bwa-mem^99^ with default parameters and removed alignments with a mapping quality score less than 30. We then conducted a case-control GWAS with the samtools-legacy program,^100^ using a likelihood ratio test to determine the association of each SNP with false gravid spot presence or absence.

To determine the appropriate significance threshold for our analysis, we used a permutation-based approach. We randomly shuffled phenotypes between individuals, re-ran the case-control GWAS analysis using the samtools-legacy program (as we had for the real data), and recorded the minimum p-value. We repeated this procedure until we had collected 500 replicates. We took the lower 5% quantile of this distribution of minimum p-values and used this value (3.7 × 10^−9^) as our genome-wide significance threshold.

#### Correcting for population structure in genome wide association study

The case-control design we used in GWAS analysis can be susceptible to false positives in the presence of population substructure. This is a particular concern with traits like the false gravid spot that may have a function important in mating (and thus be linked with assortative mating^122^). Because the data we collected is low-coverage, standard approaches to control for population structure are not well-suited for our data. Nonetheless, we implemented several analyses to explore the potential effects of population structure on our results. We first generated “pseudo-haploid” calls for each individual by generating pileup files with bcftools,^100,123^ randomly sampling a read from each position, and assigning the allele supported by that read as the allele present in that individual. If a site was not covered by any reads in an individual, it was coded as missing. After excluding sites with a minor allele frequency less than 2% or missing in 75% of individuals using plink^103^ –recode, we performed PCA analysis with plink using this data. We asked whether there was a correlation between false gravid spot phenotype and any of the first 10 PCs, which together explained ~28% of the variation in the data.

We also used these pseudo-haploid calls to repeat the GWAS with plink^103^ while explicitly accounting for population structure by including the first four PCs as covariates. Despite the expectation that we would have reduced power due to pseudo-haploid calls, we still detected an association on chromosome 2 that surpassed the genome-wide significance threshold (Figure S2). Since different iterations of an analysis using pseudo-haploid calls will differ due to stochasticity introduced by sampling, we repeated this procedure 10 times.

#### Long-read *Xiphophorus* reference assemblies

We generated a new chromosome-level *X. birchmanni* genome using PacBio long-read sequencing (Table S1). The reference individual did not have the false gravid spot phenotype and was a lab-raised fish originating from a stock derived from the Coacuilco population. Genomic DNA was isolated from tissue using the Qiagen Genomic-Tip kit. We followed the manufacturer’s protocol with slight modifications. Tissue was digested in 1.5 mL of Proteinase K and 19 mL of Buffer G2 for two hours. The sample was gently mixed by inversion every 30 minutes. The sample was applied to the column following equilibration of the column with 10 mL of Buffer QBT. The column was washed two times with 15 mL of Buffer QC, and then eluted in a clean tube with Buffer QF. DNA in the eluate was precipitated using 10.5 mL of isopropanol, mixed by gentle inversion, and then centrifuged at 4°C (5000 × g) for 15 minutes. The pellet was washed in cold 70% ethanol, re-pelleted, and then air dried after removal of the supernatant in 1.5 mL of Buffer EB. DNA was quantified on the Qubit fluorometer and evaluated for quality using the Nanodrop and Agilent 4150 TapeStation machine. Extracted DNA was sent to Cantata Genomic for PacBio library prep and sequencing on 2 SMRT cells. Reads were initially checked for quality using NanoPlot^124^ and residual adapters were removed using the HiFiAdapterFilt.sh script.^104^ We used hifiasm^105^ with default parameters to generate a phased diploid genome assembly. We then scaffolded the primary contigs using previously collected Hi-C data^41^ with YaHS^125^ to generate a chromosome-level assembly. All chromosomes were oriented and named with respect to the *X. maculatus* reference genome. Because chromosome 21 is thought to be the sex chromosome in most *Xiphophorus* species,^54^ we used minimap2^107^ to align both alternate haplotypes generated by hifiasm. We found a ~2 Mb region of highly divergent and structurally variable sequence on the distal end of chromosome 21. To represent both haplotypes (X and Y) in the assembly, which has been shown to improve variant calling performance on the sex chromosomes,^126^ we made a second copy of chromosome 21 with the divergent region replaced with the alternate haplotig. We then aligned these draft X and Y scaffolds together and masked the high homology (pseudo-autosomal) regions on the Y, as recommended.^126^

Because the *X. birchmanni* reference individual did not have false gravid spot, we sought to assemble a high-quality reference for the false gravid spot haplotype. To do so, we also sequenced one *X. malinche* × *X. birchmanni* F_1_ hybrid with false gravid spot with PacBio HiFi, with extraction methods identical to those described above. Given the false gravid spot is absent in *X. malinche*, the false gravid spot allele in the hybrid must be inherited from *X. birchmanni*. We chose to assemble a genome from an F_1_ hybrid because we expected that the divergence of *X. malinche* and *X. birchmanni* (0.5% per bp) would facilitate generating long phased blocks (N50 of untigs: 12.3 Mb), which would prove useful for examining structural differences within and between species. Extracted DNA was sent to Admera Health Services, South Plainfield, NJ for PacBio library prep and sequencing on 2 SMRT cells. We checked read quality and removed residual adapters as above and again used hifiasm to generate a phased diploid genome assembly. Based on our initial GWAS results, we blasted *kitlga* to the diploid assembly graph (untigs) to identify the *X. birchmanni* and *X. malinche* haplotypes corresponding to the candidate region. We then ran the resulting two untigs that contained *kitlga* through the ancestryHMM pipeline.^113^ One of the untigs, a 23 Mb sequence, was inferred to be 100% *X. birchmanni* ancestry based on the ancestryHMM pipeline, suggesting the majority of chromosome 2 was fully phased. We then manually replaced the homologous non-FGS haplotype in the reference with this untig based on coordinates generated using a minimap2 alignment, following the approach described above for chromosome 21. For consistency, we also reassembled previously published *X. malinche* HiFi reads^127^ using the computational pipeline described above and added previously collected Hi-C data^41^ (Table S1).

#### Annotation of reference assemblies

We annotated the new reference assemblies for *X. birchmanni* following a pipeline used for previous *Xiphophorus* assemblies. We first identified repeats using RepeatModeler.^119^ We took the output file from this process and repeat libraries from Repbase^128^ and FishTEDB^129^ were input into RepeatMasker,^120^ allowing additional transposable element sequences to be identified based on sequence similarity. These annotations were used to hard mask transposable elements and soft-mask simple repeats in subsequent annotation of protein-coding genes.

We annotated protein coding genes using a multi-pronged approach that included homology, transcriptome mapping, and *ab initio* prediction. For annotation using homology, we collected a total of 455,817 protein sequences from the following databases: the vertebrate database of Swiss-Prot (https://www.uniprot.org/statistics/Swiss-Prot), RefSeq database (proteins with ID starting with “NP”‘ from “vertebrate_other”) and the NCBI genome annotation of human (GCF_000001405.39_GRCh38), zebrafish (GCF_000002035.6), southern platyfish (GCF_002775205.1), medaka (GCF_002234675.1), mummichog (GCF_011125445.2), turquoise killifish (GCF_001465895.1) and guppy (GCF_000633615.1). We used GeneWise^130^ and exonerate to align these protein sequences to the *de novo* assembly to identify gene models via homology, using GenblastA^131^ to identify an approximate alignment region and improve required computational time.

We also took advantage of available RNA-seq data from multiple tissues from F_1_ hybrids between *X. birchmanni* and *X. malinche* to perform transcriptome mapping. The RNA-seq data was pre-processed using fastp^132^ and mapped to the *de novo* assembly using HISAT2.^111^ We processed mapping results with StringTie^112^ to generate gene models based on this data. We also used Trinity^133^ to assemble *de novo* transcriptomes from the RNA-seq data and aligned these transcripts to the assembly. These aligned transcripts were converted to gene models using Splign.^134^ Finally, we performed *ab initio* gene prediction using AUGUSTUS.^121^ The first round of AUGUSTUS training was performed on BUSCO genes, and the second round of training was performed using gene models identified from the methods described above. This database was used as ‘hints’ for AUGUSTUS gene prediction.

With these predictions in hand, we generated a final consensus annotation by screening models by locus. When two models competed for a splice site, we retained the gene model that was better supported by transcriptome data. When a terminal exon (with start/stop codon) from *ab-initio* or homology gene model was better supported by transcriptome than that of the retained gene model, the latter was replaced. We also kept an *ab-initio* prediction when its transcriptome support was 100% and it had no homology prediction competing for splice sites.

Given that automatic annotation approaches, such as those described above, can miss exons separated by large introns and thus lead to incomplete gene models, we inspected the annotation of *kitlga*. In particular, visualization of RNA-seq data aligned to the *X. birchmanni* reference genome with HISAT2, and the gene models of *kitlga* for several tissues generated by stringtie, suggested an additional first exon and UTR 30 kb upstream of the second exon, and 1.5 kb downstream of the structural variant (Figure S3). While this has implications for interpretation of the GWAS peak, for downstream analyses involving gene expression, we did not modify the transcriptome to maintain consistency with other genes in the genome (e.g., for RNA-seq analyses).

#### Analyses of protein evolution at *kitlga*

We compared amino acid sequences at *kitlga* between *X. birchmanni* individuals with and without the false gravid spot to *X. malinche*, the sister species of *X. birchmanni* which is fixed for the non-false gravid spot phenotype. We reasoned that if coding changes were involved in phenotypic differences between individuals, we would see this reflected in this analysis of amino acid sequences. We extracted the predicted exons from previously generated pseudoreference sequences for 25 *X. birchmanni* individuals^135^ and five *X. malinche* individuals.^136^ To calculate the rate of nonsynonymous substitutions per nonsynonymous site versus the rate of synonymous substitutions per synonymous site between *X. birchmanni* and *X. malinche* at *kitlga* we used the codeml program in PAML.^137^

#### Linkage disequilibrium analysis in the region implicated by GWAS

To investigate variants in the associated region identified with GWAS, we used a previously collected dataset of 23 individuals from the Coacuilco population sequenced at higher coverage (median 18.4×). Reads were mapped to the *X. birchmanni* reference genome using bwa, and realigned around indels using picardtools. We called variants using GATK4^102^ and filtered each vcf individually following guidelines outlined in the GATK best practices that we previously verified have good performance in *Xiphophorus*.^98^ We additionally filtered all SNPs within 5 bp of an indel. We then merged all SNP calls and removed sites with >10% missing data using bcftools. We used plink to calculate linkage disequilibrium (LD) for all SNPs within 1 Mb surrounding the associated SNP located in the center of the GWAS peak. We also determined the genotype of individuals in this region based on sets of SNPs that are diagnostic for the rearranged haplotype, finding two homozygote false gravid spot, eight heterozygotes, and 16 homozygous non-false gravid spot individuals.

#### Long-read sequencing and assembly of males with different false gravid spot phenotypes

For 11 of the 12 individuals collected from three populations as described above, we extracted high molecular weight DNA from brain and liver tissue with the NEB Monarch HMW DNA Extraction Kit for Tissue (Catalog #T3060S, NEB, Ipswich, Massachusetts). The purified DNA was size selected for >40kb fragments with the PacBio SRE XL buffer (Catalog #102–208-400, PacBio, Menlo Park, California). Long-read sequencing libraries were then generated by Oxford Nanopore R10.4.1 sequencing. Approximately 3 μg of size-selected DNA was prepared with the Oxford Nanopore ligation sequencing kit (Catalong #SQK-LSK114, Oxford Nanopore, Oxford, United Kingdom) following the manufacturer’s instructions, except we used half volumes of library prep reagents and extended bead elution steps from five minutes to several hours at 37°C to allow very long DNA fragments to resuspend completely. We loaded 200–300 ng of prepared library onto a R10.4.1 PromethION flow cell and sequenced on a P2 Solo sequencer with live basecalling with the fastest model enabled. After our targeted sequencing depth was achieved, the data were basecalled again with Guppy v6.4.6 (Oxford Nanopore) using the super-accuracy model. Reads passing the default quality score filter (Phred-scaled QV10) were assembled using the diploid assembler shasta^106^ with default parameters.

For the remaining individual, a male from Coacuilco, we extracted genomic DNA using the Promega Wizard HMW DNA extraction kit (Catalog #A2920, Madison, WI), following the manufacturer’s instructions. DNA was quantified on the Qubit fluorometer and evaluated for quality using the Nanodrop and Agilent 4150 TapeStation machine. Extracted DNA was sent to the University of Washington Long Reads Sequencing Center for PacBio library prep and sequencing on 1 SMRT cell. A diploid assembly was generated from the HiFi reads with hifiasm as described above.

#### Identifying diagnostic false gravid spot SNPs between *X. birchmanni* haplotypes

To identify SNPs diagnostic of false gravid spot haplotypes, we leveraged the long-read dataset collected across populations. For simplicity, we focused on the region that is inverted in false gravid spot haplotypes, since this region single copy in all haplotypes. To identify diagnostic SNPs in the region, we first mapped all haplotypes to the *X. birchmanni* non-false gravid spot reference using minimap2. We then used IGV to search the 8.8 kb inverted region for single nucleotide differences between haplotypes that were fixed between the inverted and non-inverted haplotypes. We excluded SNPs that were not fixed in either haplotype as well as SNPs within 5 bp of an indel. Together, this analysis identified 147 fixed differences between haplotypes that we use as “diagnostic SNPs” in analyses.

#### Analysis of structural variation at the false gravid spot locus

For an initial analysis of structural variation comparing the *X. birchmanni* non-false gravid spot reference and the *X. birchmanni* false gravid spot reference sequence, we performed pairwise alignments in MUMmer4.^58^ For population level analyses from long-read assemblies, we first identified phased haplotypes containing the structural variant by conducting BLASTn^138^ searches for the inverted region against the diploid assemblies (Assembly-Phased.fasta for nanopore assemblies and *utg.fa for HiFi assemblies). We chose to use these files, which are produced as part of the assembly pipelines, because they contain all the sequence information but are more conservative regarding assembly errors (untigs have fewer assembly errors than contigs^105^). We aligned these phased haplotypes to the *X. birchmanni* non-false gravid spot reference genome using MUMmer4 with default parameters and visualized the results using a custom R script. For ease of comparison between haplotypes, we also used MUMmer4 to align regions identified in the reference alignment (segmental duplications, inversion, transposable element insertions) to each phased assembly. We visualized the regions of homology using the gggenes R package (https://cran.r-project.org/web/packages/gggenes), keeping segments with 1 kb length and merging consecutive segments separated by less than 500 bp.

We initially tested several approaches to quantitatively call structural variation at the focal locus on chromosome 2 from long-read data. However, due to the complex nature of the rearrangement associated with the false gravid spot, we were limited to structural variant callers that recognize both inversions and insertions/deletions (indels). We tried variant callers that take advantage of data from both reads (e.g., Sniffles2^139^ and SVision^140^) and assemblies (e.g., PAV^91^). While these approaches always identified structural variants in false gravid spot haplotypes, the results were not consistent in the number and nature of variants called. Given this inconsistency, we report results of the MUMmer4-based analysis of structural rearrangements in this region.

#### Evaluation of DNA structural features near GWAS peak

We used available computational tools to compare the frequency of non-B DNA in the regions surrounding the false gravid spot and non-false gravid spot haplotypes using the program nBMST^141^ (https://github.com/abcsFrederick/non-B_gfa). This program outputs the coordinates of predicted features associated with non-B DNA for each fasta sequence analyzed, including A-phased repeats, G-quadraplexes, Z-DNA, direct, inverted, and mirror repeats, and STRS. We compared both the frequency and the length of these features between the phased false gravid spot and non-false gravid spot reference haplotypes, locally around the GWAS peaks identified mapping to both reference sequences.

#### Phylogenetic analysis

From each phased haplotype described above, as well as *X. malinche* and *X. hellerii*, we extracted the inversion and *kitlga* gene using a custom R script that identified the region using MUMmer4 alignments. We generated multiple sequence alignments for each region using Clustal Omega and inspected each alignment manually. From these alignments, we ran RAxML to generate maximum likelihood trees. To assess support at each node, we ran 1000 bootstrap replicates. We rooted each tree using the reroot function in phytools^108^ using *X. hellerii* as an outgroup. We then visualized the tree as a cophylo plot using phytools, where tips derived from the same phased assembly contig were connected.

#### Whole tissue RNA-seq

To explore expression differences in individuals with and without the false gravid spot, we dissected three tissues from 13 individuals: brain, body wall musculature, and the tissue surrounding the gonopodial suspensorium which consists of the erector analis major muscle and its perimysium. We extracted total RNA with the Qiagen RNAeasy Mini Kit (Catalog #74106, Qiagen, Valencia, CA) and sent RNA to Admera Health Services for library preparation using the NEBNext Ultra II Directional library prep kit with Poly A Selection. Extracted RNA was sent to Admera Health Services, South Plainfield, NJ for library preparation and sequencing on Illumina HiSeq 4000. Extractions and library preps were paired between groups (e.g., at least one false gravid and non-false gravid replicate for each extraction and library prep batch) to control for batch effects in statistical analysis downstream.

Raw reads were trimmed to remove adapter sequences and low-quality base pairs using TrimGalore!^109^ with parameters –phred33 –quality 30 -e 0.1 –stringency 1 –length 32 –paired –retain_unpaired.^142^ Trimmed reads were pseudoaligned to the *X. birchmanni* reference transcriptome with *kallisto*^61^ and the differential expression analysis was performed using DESeq2.^62^ Because the *X. birchmanni* reference transcriptome does not contain isoforms, we assumed one transcript per gene in the reference genome. We removed genes with zero counts in all samples before beginning analysis of differential expression with DESeq2. We analyzed each of the three tissue types separately and used an analysis model that modeled gene expression as a function of false gravid spot phenotype and extraction/library preparation batch. Gene counts were normalized by library size, and we used the “local” function in DESeq2 to estimate within-group dispersion. We tested for significant differences in expression using the Wald test and calculated shrunken log-fold changes in expression using the ashr package.^110^ We consider genes with an adjusted p-value<0.05 between groups to be differentially expressed.

#### Allele specific expression of kitlga in lab-generated hybrids heterozygous for the false gravid allele at kitlga

Based on our RNA sequencing results, we wanted to determine if differential expression of the *kitlga* allele associated with the false gravid haplotype was driven by changes in *cis*. Because there was little sequence variation within *X. birchmanni* in the *kitlga* coding region, but several fixed synonymous differences between *X. birchmanni* and its sister species *X. malinche* (Figure S9), we conducted this experiment using hybrids between the two species. Because *X. birchmanni* is polymorphic for the false gravid spot whereas *X. malinche* is fixed for its absence, we used early generation hybrids (F_2_-F_3_) and selected individuals with the false gravid spot. We confirmed by genotyping that these individuals were heterozygous for *X. birchmanni* and *X. malinche* ancestry in the region surrounding *kitlga* using the AncestryHMM^113^ pipeline.

We designed primer sets for pyrosequencing using the Qiagen Pyromark software. We tested several primer sets on three tissues (brain, gill, testis) derived from pure *X. birchmanni* and pure *X. malinche* cDNA. Briefly, we amplified samples (N=3 of each species) using the PyroMark PCR kit (Catalog #978703, Qiagen, Germantown, MD) following the manufacturer’s instructions. PCR reactions were submitted to the Protein and Nucleic Acid facility (Stanford University, CA) for pyrosequencing. We analyzed our results using the Qiagen Pyromark software. Based on the results of these quality tests, we selected three primers that performed well (>95% support for the expected allele in all individuals of both parental species; Table S4). We repeated this approach to collect data from seven hybrids with the false gravid spot in four tissues: brain, gill, testis, and erector analis major and its perimysium. For each tissue, we calculated the ratio of the false gravid spot (*X. birchmanni*) allele to the non-false gravid spot allele (*X. malinche*).

#### Developmental timing of false gravid spot in *X. birchmanni*

To characterize the development of the false gravid spot, we tracked fish from the onset of puberty until several months after sexual maturity. We established two 115 L tanks of newborn *X. birchmanni* fry from lab stocks. We expected these fish to be segregating for the false gravid spot based on the observed phenotypes in the adult stocks. We set up tanks with ~25 fry and maintained them following normal husbandry procedures until they reached 4–5 cm of length. Once fish reached this size, they were marked with a unique color combination using elastomer injection tags (Northwest Marine Technologies). Fish were checked weekly for evidence of gonopodial differentiation. As soon as males could be unambiguously determined based on thickening of the anterior segments of anal fin ray 3, they were separated into a grow-out tank and photographed weekly. Coincident with weekly photographs, we also examined males under the dissection scope and scored the stage of gonopodial development (Figure S10), ranging from thickening of the 3^rd^ ray (onset of external phenotypic sexual differentiation) to a fully mature gonopodium capable of fertilization with differentiated distal structures including a claw, blade, hook, spines and serrae (Figure S10; Table S4).

To understand if the presence of the false gravid spot influenced the development of other secondary sexual traits, including morphological and pigmentation traits, we ran a principal components analysis on adult males in this dataset using the prcomp function in R. We considered measurements of standard length, body depth, longest dorsal ray, and dorsal length as morphological variables. For pigmentation, we considered binary presence versus absence of xanthophore pigmentation in the dorsal and caudal fin, melanic pigmentation in the vertical bars, horizontal line, spotted caudal, and carbomaculatus.

#### *X. birchmanni* population demography and simulations of polymorphism maintenance

To better understand the demographic history of *X. birchmanni* populations, we mapped nanopore reads to the reference *X. birchmanni* assembly using minimap2 -x mapont and called variants using longshot,^114^ a variant caller designed for single-molecule reads with higher error rates than Illumina reads, with default parameters. To infer demographic history for each population, we ran PSMC^69^ using longshot variants for each sample. To scale the output, we used 3.5 × 10^−9^ as the mutation rate and 0.5 years for generation time, following previous work.^98^

To explore the likelihood of maintenance of a neutral polymorphism in simulated populations matching the inferred demographic histories, we performed forward-time population simulations with SLiM.^70^ We tracked evolution at a single site, prevented recurrent mutations at that site, and directed the program to track substitutions as well as polymorphisms. In generation two, we used the genomes.addNewDrawnMutation function to establish a polymorphism at the tracked site at the expected allele frequency based on observed phenotype frequencies in each population (Benito Juarez: 0.13, Izapa: 0.23, Coacuilco: 0.29). We took the estimated N_e_ for each time segment from PSMC from a single individual from each population. Then, we implemented population size changes at time segment intervals output by PSMC using setSubpopulationSize to make the simulated population exactly match the inferred N_e_ at that timepoint. Due to differences in population history, we lose resolution in demographic inference at different time periods. For Izapa, which has undergone a strong bottleneck, we lose resolution in PSMC inference ~65k generations in the past. Although PSMC inference for Coacuilco and Benito Juarez extends to deeper timescales, we selected the starting time segment for these populations that most closely matched where we lose resolution in Izapa to begin simulations for each population. This ranged from 63–73k generations in the past for Coacuilco and Benito Juarez. After initializing the simulation, we allowed evolution to occur until the focal polymorphism fixed or was lost or until the simulation ran to completion. Due to well-known artifacts in PSMC inference in recent time points, the last four time segments, constituting 289 generations, were excluded from the simulations. We recorded how frequently the polymorphism was retained in each population across 10,000 replicate simulations for each population.

#### Detecting genomic signatures of balancing selection

To pursue independent lines of evidence that the false gravid spot is maintained by balancing selection, we investigated patterns of genomic diversity near the false gravid spot locus. Because only a small number of individuals were sequenced with long-read approaches, and because combining data across populations can generate artifacts in these analyses due to population structure, we again used previously generated short-read data for 23 individuals from the Coacuilco population. An additional benefit of using these individuals is they were collected randomly with respect to false gravid spot phenotype, and therefore represent a less biased sample of the population. Due to potential technical issues with mapping and variant calling short-read data near and within the copy number variable regions within the structural variant, we focused on the 8.8 kb chromosomal inversion. The inversion was present and single-copy in all haplotypes analyzed with long-reads, averting issues with mappability driven by copy number variable regions. Using variant calls generated as described above, we first calculated mean nucleotide diversity (π) using pixy^115^ in 8.8 kb windows across chromosome 2. We next used vcftools^101^ to calculate Tajima’s D within the inversion and compared it to windows of the same length chromosome-wide. Lastly, we used the R package balselR to calculate NCD1 statistics^73^ in windows chromosome-wide of the same size. For this analysis, we used a target frequency of 0.3, based on the empirical observation of allele-frequency at the FGS locus, but found that in practice the NCD1 results were not sensitive to using different target frequencies. We compared the value of the inversion to all windows across chromosome 2, and also only to windows equal or lower than the estimated recombination rate of the inverted region: 7.595 × 10^−5^ ρ/bp a value in the 8^th^ percentile genome wide.

#### Frequencies across and within *X. birchmanni* populations

To evaluate variation in the frequency of the false gravid spot in *X. birchmanni*, we visited nine populations across the species range. We phenotyped adult males collected using baited minnow traps for presence or absence of the false gravid spot in the field. To understand how much false gravid spot frequency varies over time, we also sampled phenotypic frequencies from the Coacuilco *X. birchmanni* population from 2017–2023. Again, we phenotyped only adult male fish for the false gravid spot.

#### Male-male interaction open arena behavioral experiments and analysis

To test if the presence of false gravid spot affects male-male interactions, we set up triads consisting of one large focal male (n=20), and two sized matched small target males—one with false gravid spot and one without—in 40L (50.8 × 25.4 × 30.5 cm) open-field arenas. All target males lacked sexually dimorphic traits that typically develop later in life. All arenas were equipped with two half terracotta pots for shelter and all sides of each arena except the front and top were covered on the exterior with black plastic for visual occlusion. The interactions of each focal male were video recorded from the front for 15 minutes (GoPro Hero10 camera) twice daily for four consecutive days. Four trials were run concurrently, and the experiment lasted for five weeks. The day before the trial, a dominant male and female were added to a new tank to habituate for at least 16 hours. The first 15-minute video was recorded in the morning within five minutes after introducing the two size-matched, randomly selected stimulus males to the trial arena. The second video was recorded five to six hours later. Following this recording, stimulus males were removed from the tank and each focal male was moved to a new arena with a new female to habituate overnight before the next trial. This procedure was followed to ensure that no focal male saw the same targets twice and no target male was used more than once in each week.

The first five minutes of each 15-minute video was treated as an acclimation period and discarded. Behaviors were then scored for the remaining 10 minutes of each video. This resulted in a total of 80 minutes of observation per focal male across trials. The following behaviors were scored from recorded videos with the aid of computer assisted event logging software (BORIS^117^ v.7.13.9): number of chases – rapid movement by focal male toward a target resulting in the target immediately swimming away; number and duration of sustained chases – chases where focal male continued to pursue target after initial interaction; number and duration of aggressive displays – characterized by dorsal flaring while facing in the opposite direction (head to tail) often accompanied by tail beating and nipping behaviors; number and duration of courtship bouts – side-by-side dorsal flaring and circling of target by focal male; gonopodial flexes – the rotating forward of the gonopodium, a behavior associated with ejaculation preceding mating attempts in poeciliid fish; number of approaches – when the focal male swims to within one body length of a target individual without chasing or engaging in either courtship or aggressive displays; time spent hiding. Total time spent chasing was calculated by multiplying the total number of non-sustained chases by 0.5 seconds, the typical length of such interactions, and adding the duration of sustained chases. All measures except hiding were categorized by the target to which the focal male was directing its attention.

#### Dichotomous trial behavioral experiments and analysis

To test if females displayed a preference or disdain towards the false gravid spot, we ran a series of dichotomous choice tests using animations of male *X. birchmanni*. Females originated from two lab stocks from the Coacuilco population: a wild-caught cohort (n=17) and a cohort born in the laboratory (n=23). Each trial was run for 55 minutes. The animations (Figure 5D) were of two types: (1) paired unornamented males with and without false gravid spot and (2) paired ornamented males with and without false gravid spot. Animations were generated using models based on images of adult males without the false gravid spot, and a false gravid spot was digitally added to the relevant animation. Animations were made using blender and scaled to be the same standard length (based on the average adult standard length in the Coacuilco population). To control for side bias, after a five minute rest period we displayed identical animations with the location of the false gravid spot male on the opposite monitor. Each trial animation was five minutes long, and in total each female saw four videos (differing in ornamentation and side of the stimulus). Fish were randomly shown one of four possible trial orders. The rest and acclimation periods showed a screen with the tank background but no fish animation stimulus.

We used EthoVisionXT16^116^ to track movement of fish during the female preference behavioral trials with color marker tracking. We chose a marker color range for subject identification that was as narrow as possible while still picking up fish in all areas of the tank. We created arena settings that encompassed the full tank as much as possible, while excluding anything that fell into the marker color range, such as shelters, the edges of the tank, and small reflections on the water. We created two preference zones on each side of the tank, one 25 cm from the video screen, and a closer nested zone 5 cm from the video screen. The center of the tank was the neutral zone. After tracking acquisition at a sample rate of 7.73 samples per second, we used EthoVision’s track editor feature to manually correct the location of the tracked fish as needed. This primarily occurred when the fish was on the very edge of the tank or within the shelter. We defined the start of a trial after the female visited both zones. We analyzed the difference in proportion of the trial spent in association with either stimulus.

#### Power analyses of dichotomous mate choice trials

Dichotomous choice trials are intended to measure the strength and direction of female preference for a given signal. In practice, differences detected in preferences for different stimuli or between different groups of females in behavioral trials could reflect true differences in the strength of preference, or instead point to differences in power between experiments. To explore these possibilities, we performed simulations to determine our power to detect preferences of various effect sizes given our experimental design and sample size. We calculated difference in preference for each individual and then determined the mean, standard deviation, and sample size for each of the 4 groups. These groups were: 1) lab-born females tested against ornamented stimuli, 2) lab-born females tested against unornamented stimuli, 3) wild-caught females tested against ornamented stimuli, and 4) wild-caught females tested against unornamented stimuli. For each scenario, we then generated simulated data for each group using a range of effect sizes for preference (from 0 to 0.4 in increments of 0.025). Specifically, here effect size refers to the difference in the proportion of time a female spends with the two stimuli. As an example, to generate an effect size of 0.4, a hypothetical female could spend 60% of the trial associated with the non-false gravid animation, 20% with the false gravid animation, and 20% with neither stimulus. To generate the simulated data, we assumed a normal distribution with the mean set to the focal effect size and used the observed standard deviation from the trial of interest and the sample size from the trial of interest. In each simulated dataset, we determined whether the estimated effect size differed from zero using a Wilcoxon test at p<0.05, as we had in the analysis of the empirical data. To calculate expected power for each effect size and for each group, we calculated the proportion of tests where we detected an effect out of 10,000 replicate simulations.

#### Scototaxis trial behavioral experiments and analysis

To study if the false gravid spot influenced boldness, we performed a scototaxis assay in males born in the laboratory. Fish typically prefer a dark tank background and time spent on a white background is a routinely used measure of boldness.^143,144^ We constructed trial lanes (50 cm L × 19 cm W × 19 cm H) and then lined one half with white and one half with black custom cut matte pvc foam board to reduce reflectivity. We rinsed the trial lanes between experiments and performed a water change. We recorded videos from above for a trial duration of five minutes and used EthoVisionXT16^116^ to track movement of fish. Time spent in the white zone and latency to enter the white zone were scored as measures of boldness. To control for side bias, we tested each fish twice with the zones flipped and took the mean value of the amount of time spent on the white or black background across the two trials. We kept the standard ethovision experiment settings mode and contour-based center-point detections for this set of trials. We chose the differencing detection mode with a fixed background image and the setting for the subject darker than the background, which accurately tracked the fish when in the white zone. Because the fish was not reliably detectable when in the dark zone, we designated that half of the tank as a “hidden” zone in the Arena settings. Whenever the fish entered the hidden zone, its position was assumed to be at the center of the zone. We created a tracking zone in the white zone but left an approximately 1 cm gap between the edge of the hidden zone and the white zone so that fish were not counted as entering the light zone until the majority of their body had left the hidden zone. After tracking acquisition at a rate of 6.25 samples per second, we used EthoVision’s track editor feature to manually correct the location of the tracked fish as needed.

#### Field observations

Using snorkels and masks, we conducted focal observations in the Río Coacuilco. After selecting an area where there was at least one *X. birchmanni* male, we waited for at least three minutes for the fish to habituate to our presence. After the habituation period we counted the numbers of males (identified by the presence of a gonopodium) and females (identified by the presence of a gravid spot and absence of a gonopodium), within one square meter of the focal male. We scored males for the presence of the false gravid spot, and, as covariates, we also scored the focal male for body size and size of dorsal fin. Because we were unable to directly measure the fish, we scored size as either smaller or larger than 44 mm reference line, based on the average male standard length for this population. We also recorded dorsal fins as either “small” or “large” for their body size. We observed males for five minutes or until the male swam out of the area, remaining at least 1 m away from the focal male when possible. Each observer switched between watching a male with and without false gravid spot in the same location and moved to a new area in the river between these pairs of observations to avoid repeated measures of the same males. We recorded number of courtship displays performed by the focal male toward a female, number of gonopodial thrusts (i.e., attempted copulations), number of times the focal male chased and did aggressive displays to another male (i.e., aggressive behaviors), number of times the focal male was chased (i.e., retreats), and number of nips at the substrate by the focal male (feeding).

#### Growth rates of juvenile males with and without the false gravid spot

We investigated if growth rates differed as a function of false gravid spot phenotype during juvenile development in natural populations. To determine growth rates from wild-caught males from the Coacuilco population, we obtained ring counts from otoliths, the structures present in the inner ears of *X. birchmanni* males. Otoliths rings in juvenile *Xiphophorus* are deposited daily and are visually distinguishable from previously deposited rings under a microscope until males reach sexual maturity, at which point the otolith growth is dramatically reduced. Thus, we reasoned that otolith ring counts from juveniles could allow us to estimate the approximate age of each fish and thus calculate their growth rates. Otoliths from 48 wild-caught juvenile males with and without false gravid spot from Coacuilco were collected and preserved in 95% ethanol. The otoliths from both the right and left side of the head were removed and mounted to a microscope slide using permount (FisherScientific). The asteriscus (middle-sized otolith) was examined because this is the most translucent of the three otoliths. The otoliths were then imaged using a Nikon ECLIPSE Ti Series confocal microscope. At 40x magnification, we manually focused on the mid-plane of the otolith and took a Z-stack of 51 images at 0.5 micron slices. We then post-processed the image stacks two ways using Fiji. First, we compiled a max projection of each image stack, sharpened once, and enhanced contrast by 0.35% (Figure S14A). Second, we selected a single image from each stack and sharpened it once (Figure S14B). To improve accuracy and reduce subjectivity, four researchers manually counted the rings of each otolith, using the cell counter plug-in in Fiji to track the rings using a color-coordinated system. Each of the four observers sequentially counted rings, moving the cell count marks until consensus was achieved. Because the two image types produced similar counts with a weaker correlation than expected, we took the mean of two counts for each otolith as an age estimate in days.

### QUANTIFICATION AND STATISTICAL ANALYSIS

Statistical details of all experiments are reported in the methods, results, and figure legends. Unless otherwise noted, mean and 2 SEM are used to summarize the data, and a false discovery threshold of 0.05 is used. For population genetic analyses, the real data was compared to simulated data (e.g., to determine the GWAS significance threshold) or to empirical distributions (e.g., for π in the inversion). Non-parametric statistical tests were used when data violate the normality assumption.

For the male-male interaction experiments, unless otherwise stated, means across all videos recording the focal male were used in downstream analyses (i.e., the four triads of which a given focal male was a member; eight observations total per focal male). Because the male-male interaction experiment, scototaxis, and female preference experiments all yielded data that deviated from expectations under a normal distribution, we used non-parametric Wilcoxon signed rank tests for all analyses comparing males with and without false gravid spot.

For behavioral observations conducted in the wild, we performed all statistical analyses using Generalized Linear Models (GLMs) with a normal probability distribution and an identity link function. After building initial models that included all independent variables, we performed a model reduction using Akaike’s information criterion corrected for small sample size (AICc), while retaining the variables with significant effects (assessed by their *P* values) and until we found a minimal model. In cases where the normality assumption of residuals was not fulfilled, we used a different probability distribution and link function until a normal distribution of residuals was confirmed. To simplify analyses, we used a single measure of the grouped behaviors (first principal component; PC1) calculated from factor analyses that included chases and displays to males (aggression) and displays and attempted copulations to females (courtship). In these cases, the full models included the three morphological traits and their interactions as factors, but also the number of males nearby (in the GLM of aggression behaviors) and females nearby (in the GLM of courtship behaviors) as covariates.

To test if there were morphological correlates of the false gravid spot in the wild, we used linear models to assess if these traits were affected by false gravid spot phenotype and sample year. We initially fit a full model containing both false gravid spot phenotype and sample year, but in our final analysis we dropped sample year since we did not find that it was correlated with morphology (p>0.05).

## Supplementary Material

supplementary material

## Figures and Tables

**KEY RESOURCES TABLE:** 

REAGENT or RESOURCE	SOURCE	IDENTIFIER

Deposited data		

NCBI Sequence Read Archive: long-read WGS data, short-read WGS data, RNA-seq data	This paper	PRJNA1043674
Dryad: image data, long-read assemblies	This paper	https://doi.org/10.5061/dryad.w6m905qxc
NCBI Sequence Read Archive: short-read WGS data	Powell et al.^41^	PRJNA610049
NCBI Sequence Read Archive: short-read WGS data	Schumer et al.^98^	PRJNA361133
Github: code used for analyses	This paper	https://zenodo.org/doi/10.5281/zenodo.13329118

Experimental models: Organisms/strains		

*Xiphophorus birchmanni* wild-caught samples	This paper	N/A
*X. malinche* x. *birchmanni* laboratory-generated hybrids	This paper	N/A

Oligonucleotides		

Primers for pyrosequencing (see Table S3)	This paper	N/A

Software and algorithms		

bwa	Li and Durbin^99^	https://bio-bwa.sourceforge.net/
samtools-legacy	Lietal.^100^	https://github.com/lh3/samtools-legacy/blob/master/samtools.1
vcftools	Danecek et al.^101^	https://vcftools.github.io/index.html
bcftools	Danecek et al.^101^	https://samtools.github.io/bcftools/bcftools.html
GATK4	McKenna et al.^102^	https://gatk.broadinstitute.org/hc/en-us
plink	Purcell etal.^103^	https://www.cog-genomics.org/plink/
HiFiAdapterFilt	Sim etal.^104^	https://github.com/sheinasim/HiFiAdapterFilt
hifiasm	Cheng et al.^105^	https://github.com/chhylp123/hifiasm
shasta	Shafin et al.^106^	https://github.com/paoloshasta/shasta
minimap2	Li^107^	https://github.com/lh3/minimap2
MUMmer4	Marcais et al.^58^	https://github.com/mummer4/mummer
phytools	Revell^108^	https://github.com/liamrevell/phytools
RAxML	Stamatakis^60^	https://github.com/stamatak/
TrimGalore!	Bolger et al.^109^	https://github.com/FelixKrueger/TrimGalore
kallisto	Bray et al.^61^	https://github.com/pachterlab/kallisto
DEseq2	Love et al.^62^	https://bioconductor.org/packages/release/bioc/html/DESeq2.html
ashr	Stephens^110^	https://github.com/stephens999/ashr
HiSAT2	Kim etal.^111^	https://daehwankimlab.github.io/hisat2/
StringTie	Pertea et al.^112^	https://ccb.jhu.edu/software/stringtie/
ancestryinfer	Schumer et al.^113^	https://github.com/Schumerlab/ancestryinfer
longshot	Edge and Bansal^114^	https://github.com/pjedge/longshot
PSMC	Li and Durbin^69^	https://github.com/lh3/psmc
pixy	Korunes and Samuk^115^	https://github.com/ksamuk/pixy
balselR	Bitarello et al.^73^	https://github.com/bitarellolab/balselr
SLiM	Haller and Messer^70^	https://messerlab.org/slim/
EthovisionXT	Noldus et al.^116^	https://www.noldus.com/ethovision-xt
Boris	Friard and Gamba^117^	https://www.boris.unito.it/
Fiji/ImageJ	Schindelin et al.^118^	https://imagej.net/software/fiji/
RepeatModeller	Flynn etal.^119^	https://www.repeatmasker.org/RepeatModeler/
RepeatMasker	Smit and Green^120^	https://www.repeatmasker.org/
Augustus	Stanke et al.^121^	https://github.com/Gaius-Augustus/Augustus

## Highlights

- The false gravid spot is a sexual mimicry polymorphism in swordtail fish
- Non-coding structural variation increases expression of *kitlga* to drive the spot
- Several lines of evidence suggest balancing selection maintains the polymorphism
- The false gravid spot alters social interactions with both males and females
