# Supplementary material for: Structural genomic variation and behavioral interactions underpin a balanced sexual mimicry polymorphism
Source: Curr Biol. Author manuscript; Available in PMC 2025 Jun 2. (PMC12129486; doi:10.1016/j.cub.2024.08.053)
Supplement: supplementary material [file NIHMS2076321-supplement-supplementary_material.pdf]

Current Biology, Volume *34*

Supplemental Information

Structural genomic variation and behavioral interactions underpin a balanced sexual mimicry polymorphism

Tristram O. Dodge, Bernard Y. Kim, John J. Baczenas, Shreya M. Banerjee, Theresa R. Gunn, Alex E. Donny, Lyle A. Given, Andreas R. Rice, Sophia K. Haase Cox, M. Luke Weinstein, Ryan Cross, Benjamin M. Moran, Kate Haber, Nadia B. Haghani, Jose Angel Machin Kairuz, Hannah R. Gellert, Kang Du, Stepfanie M. Aguillon, M. Scarlett Tudor, Carla Gutiérrez-Rodríguez, Oscar Rios-Cardenas, Molly R. Morris, Manfred Schartl, Daniel L. Powell, and Molly Schumer

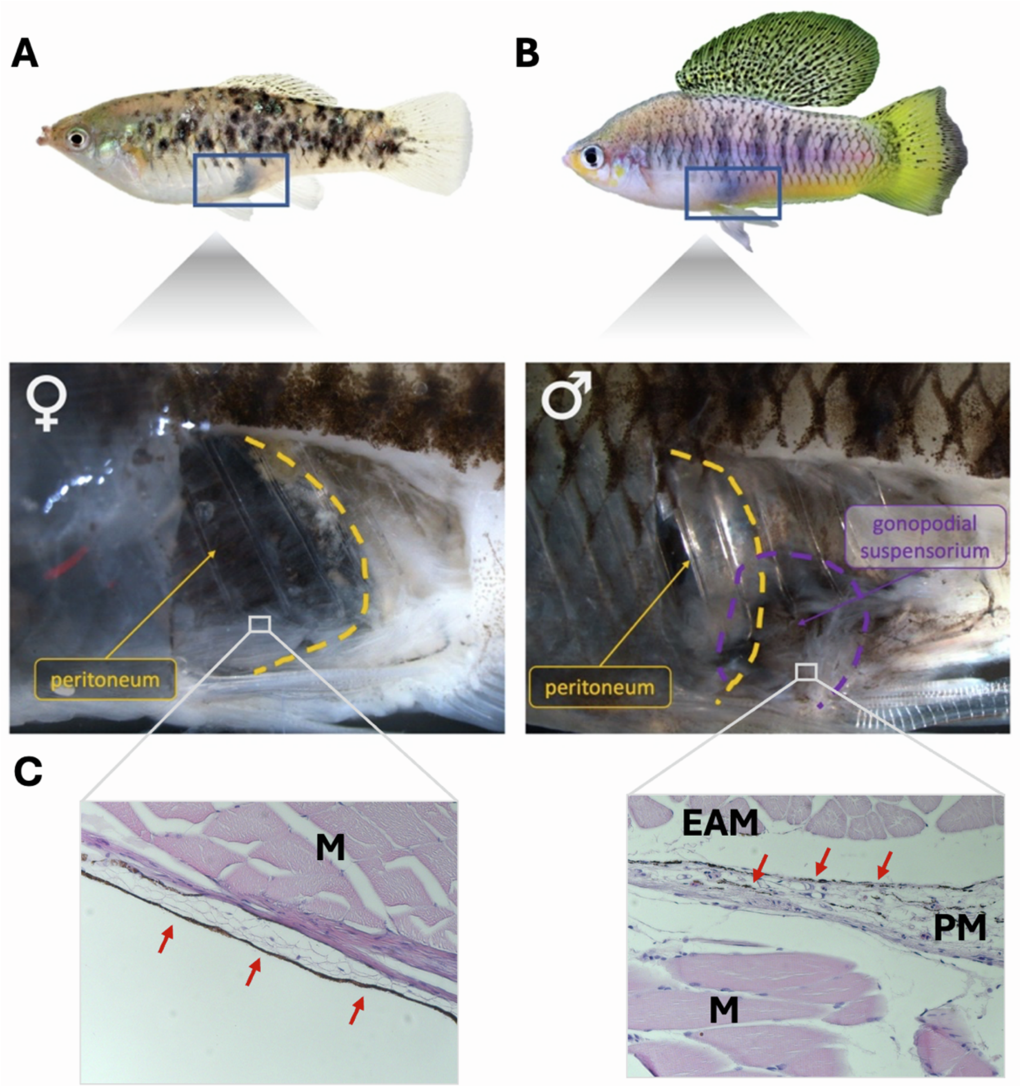

Figure S1. Anatomy of the false gravid spot, related to Figure 1. Dissections of a female **(A)** and male **(B)** *X. birchmanni* reveal the gravid spot is a distinct structure from the false gravid spot. The gravid spot in females is caused by the pigmentation of the peritoneum, whereas the false gravid spot in males occurs posterior to the peritoneum and is due to pigmentation of the tissue surrounding the gonopodial suspensorium. **C**) 20X magnification of pigmented tissue contributing to gravid spot (left) and false gravid spot (right). Labels: M – body wall musculature; EAM – Erector analis major; PM – Perimysium of EAM; E – Embryo; red arrows – pigmented melanophore cells.

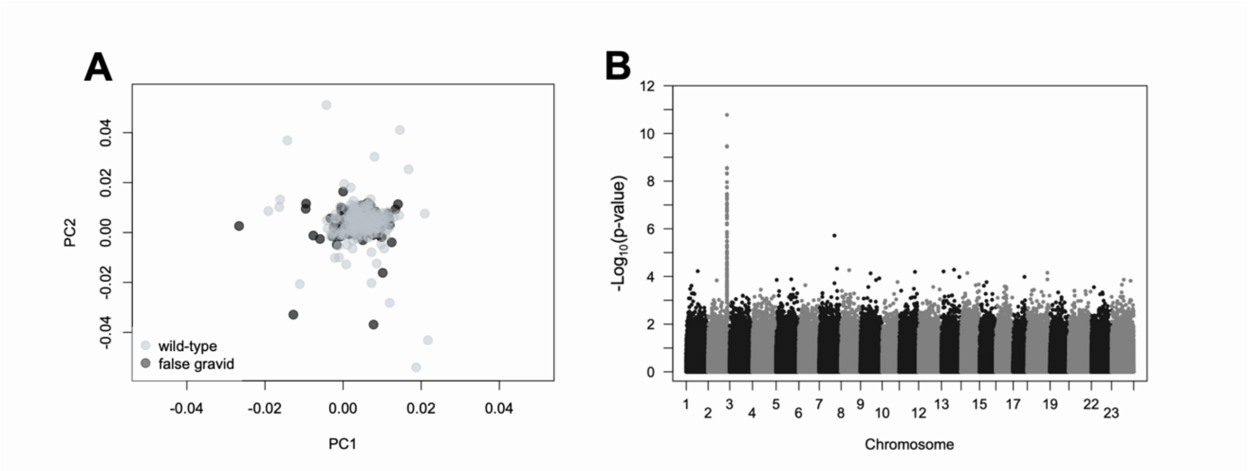

Figure S2. Correcting for population structure does not change GWAS results, related to Figure 1. **A)** We found no significant association between the false gravid spot and any of the first 10 principal components in an analysis of genome-wide SNP variation. Plotted here are PC1 and PC2 with false gravid phenotypes highlighted (non-false gravid spot –light gray, false gravid spot – dark gray). We tested for correlations between phenotype and PC for the first ten PCs (see main text). **B)** Manhattan plot showing the results of a genome-wide association study of false gravid spot phenotype using pseudohaploid calls and accounting for PCs 1-4 as covariates in the analysis using plink. Although we have lower power in this analysis due to the use of pseudohaploid calls (see main text), we still detect the strong signal on chromosome 2 upstream of *kitlga*.

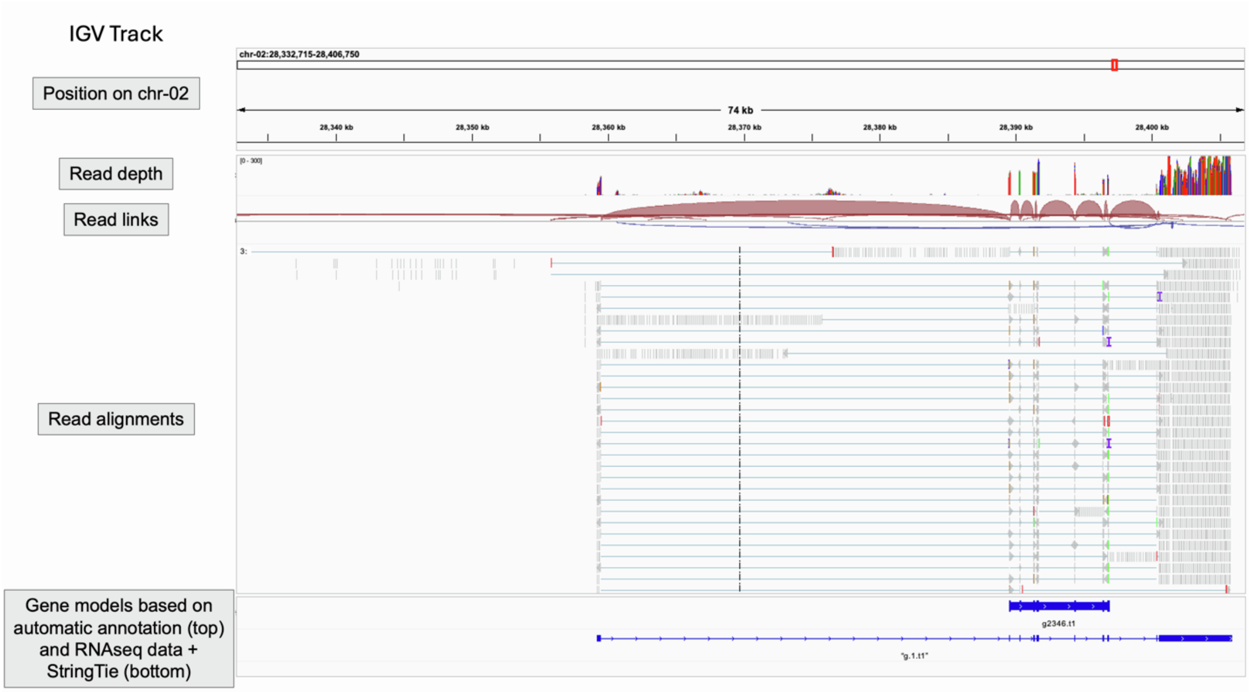

Figure S3. Annotation of *kitlga* with RNAseq data, related to Figure 1. Identification of *kitlga* exons based on RNAseq data mapped to the *X. birchmanni* reference genome with HISAT2. IGV snapshot showing alignment of RNAseq data from an F_1 individual that was used to annotate the reference genome. Top track shows the position on chromosome 2. The second track shows read depth, with the first putative exon achieving >100x read coverage per base-pair. The third track shows links between reads. The fourth track shows individual RNAseq reads mapped to the reference, with split alignments connected by blue lines. The fifth track shows results of the automatic annotation pipeline described in main text (top) as well as the results of our manual annotation described in the main text, based on the alignment and supported by gff files generated by StringTie (bottom). Note that an additional *kitlga* exon is supported by the RNAseq data and is included in our manual annotation.

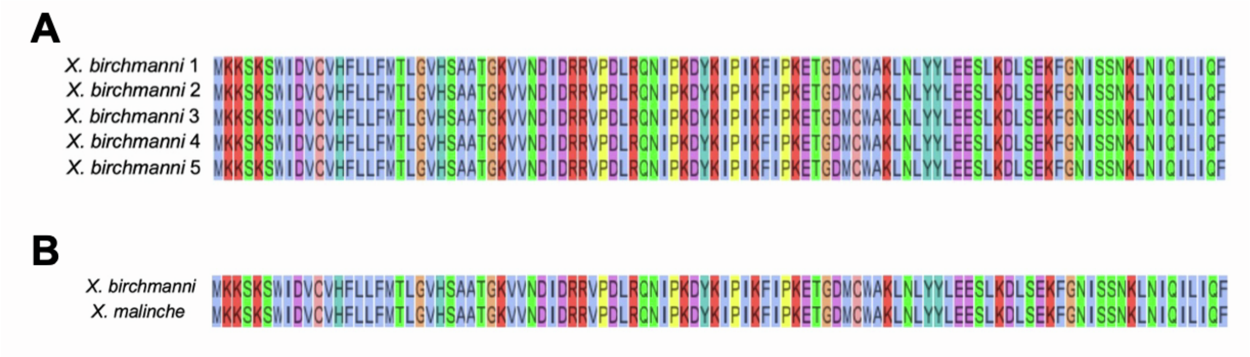

Figure S4. *kitlga* amino acid sequence is identical within and across related species, related to Figure 1. A) Alignment of *kitlga* amino acid sequences from all available *X. birchmanni* pseudoreferences with complete sequence information using clustal-omega. Individuals with masked or missing basepairs in the coding sequence of *kitlga* were excluded from visualization here but were included in the analysis of amino acid sequence similarity across individuals. **B**) Alignment of *kitlga* amino acid sequences from *X. birchmanni* and *X. malinche* reference sequences generated using clustal-omega. No nonsynonymous differences in *kitlga* sequence are detected between the two species.

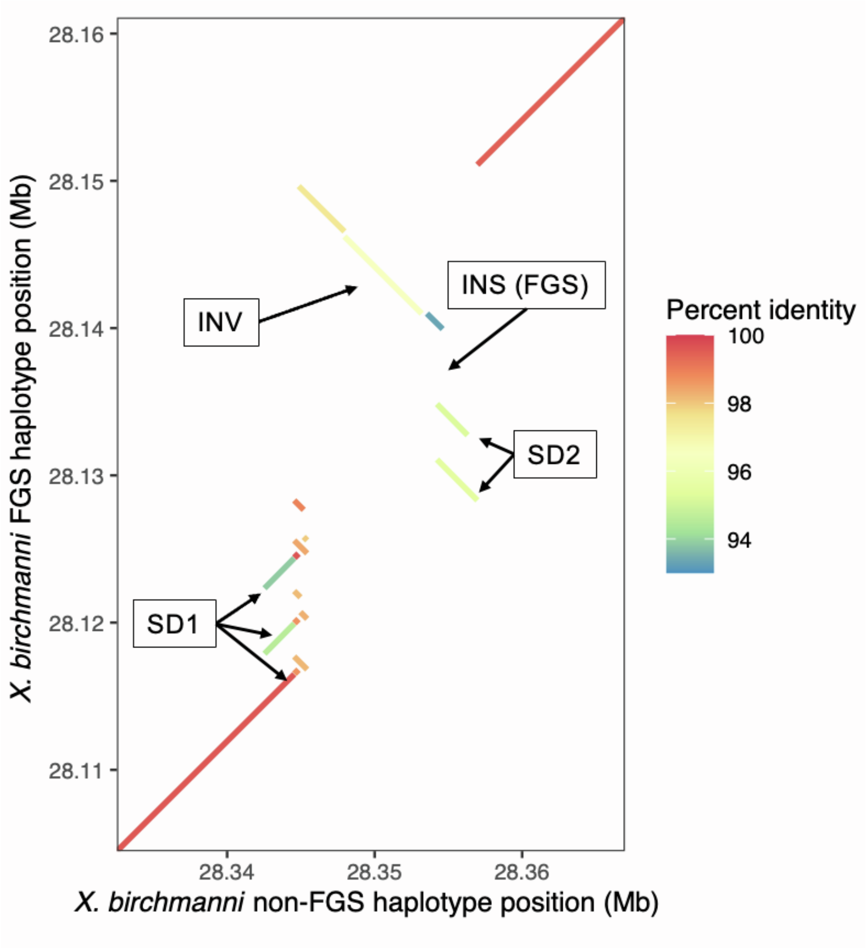

Figure S5. Structural rearrangements and divergence between reference haplotypes, related to Figure 2. MUMmer4 alignment of reference false gravid spot (FGS) and non-false gravid spot (non-FGS) haplotypes from *X. birchmanni*. Segments are colored by percent identity between the haplotypes. Individual components of the structural variant are labelled. SD1 - segmental duplication 1; SD2 - segmental duplication 2; INS (FGS) - insertion in false gravid spot haplotype (piggyback 4 element); INV - inverted region between false gravid spot and non-false gravid spot haplotype. INS (non-FGS) - insertion in non-false gravid haplotype (INDELs are visualized as a gap in one alignment or the other).

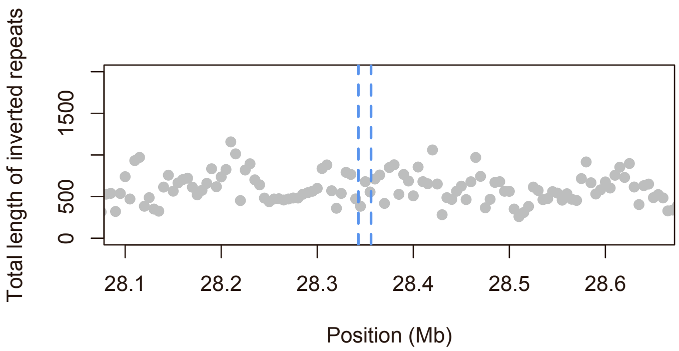

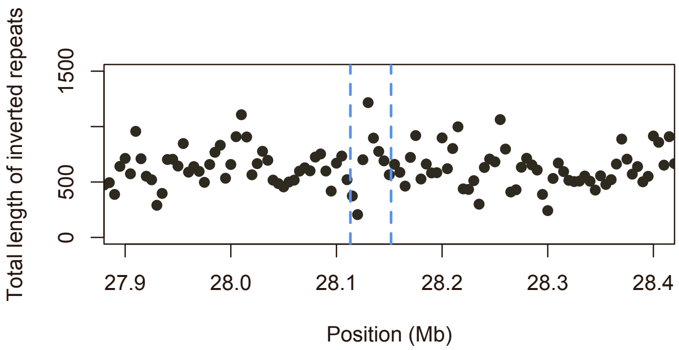

Figure S6. Abundance of non-canonical DNA structures near false gravid spot locus, related to Figure 2. Comparison of the length of inverted repeat sequences in sliding 5 kb windows identified by nBMST between the non-false gravid spot (top) and false gravid spot (bottom) haplotypes. Inverted repeats are enriched in the false gravid spot haplotype (bottom, black) compared to the non-false gravid spot reference sequence (top, grey). Significant regions in the GWAS analysis based on both reference haplotypes are noted by the blue dashed lines. Each dot represents the length in basepairs of the 5 kb region attributable to inverted repeats.

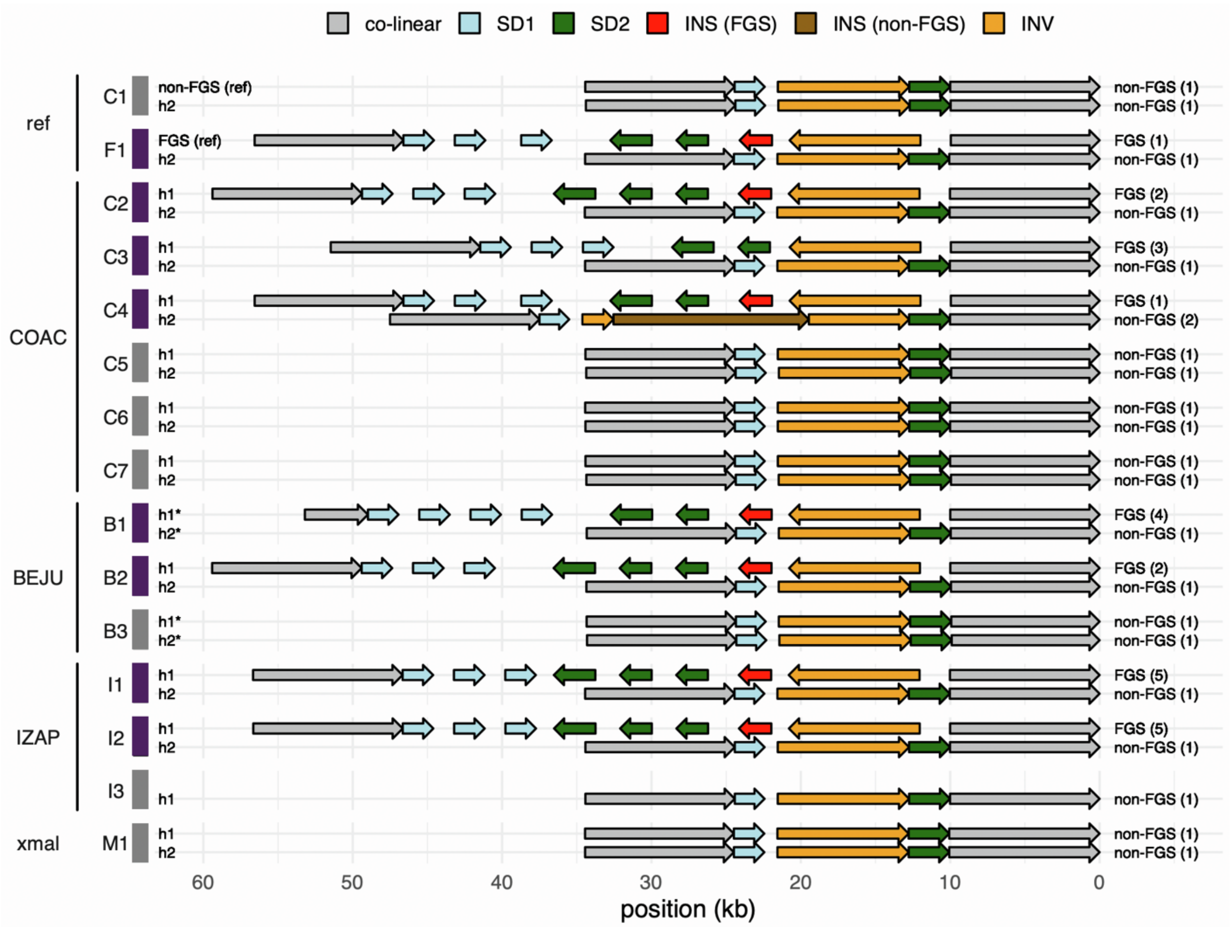

Figure S7. Structural haplotypes organized by individual, related to Figure 2. Cartoons of structural variation in haplotypes generated for this study, based on MUMmer4 alignment coordinates for each individual. Haplotypes shown here are the same as Figure 2b, with the addition of a pure *X. malinche* individual. Populations and species are denoted on left (reference individuals – ref; *X. birchmanni* Coacuilco – COAC; *X. birchmanni* Benito Juarez – BEJU; *X. birchmanni* Izapa – IZAP; *X. malinche* – xmal). Alpha numeric labels on left denote individuals, with the first letter corresponding to population, and the number denoting the sample (e.g. B1– Benito Juarez individual 1). Note that reference individual "ref F1" is an F_1 hybrid between *X. birchmanni* x *X. malinche*, so F1_h2 is a *X. malinche*-derived haplotype. Colors on left indicate false gravid spot (FGS) phenotype of the focal individual, with purple denoting false gravid spot and gray denoting non-false gravid spot phenotypes. All individuals with the false gravid spot phenotype have one rearranged haplotype compared to the reference genome, and one haplotype that is largely colinear. All individuals without the false gravid spot (including X. malinche) have 2 haplotypes colinear with the reference. SD1 - segmental duplication 1; SD2 - segmental duplication 2; INS (FGS) - insertion in false gravid spot haplotype (piggyback 4 element); INS (non-FGS) - insertion in non-false gravid haplotype); INV - inversion. Labels on right correspond to haplotype class, with variant alleles numbered.

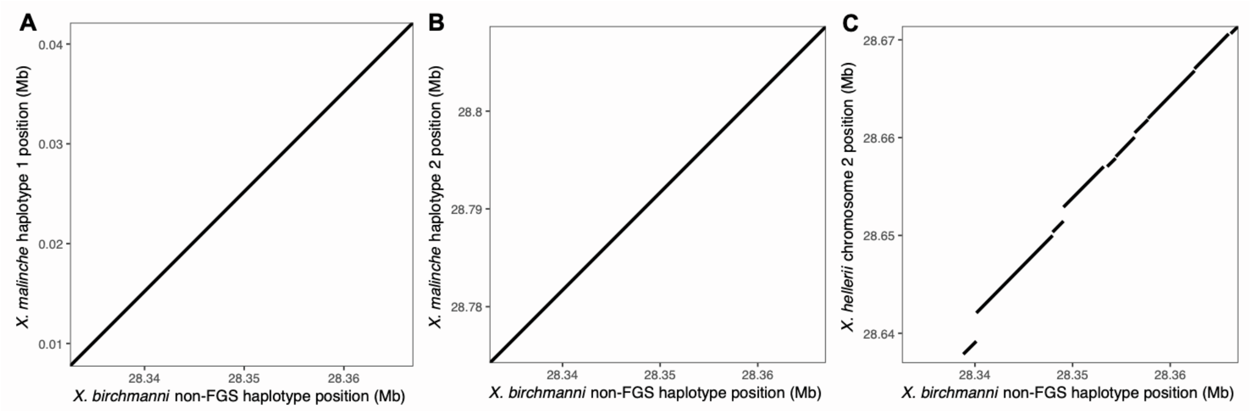

Figure S8. Co-linearity between non-false gravid spot haplotypes across species, related to Figure 2. A) MUMmer4 alignment of non-false gravid (non-FGS) spot *X. birchmanni* haplotype with haplotype 1 from the *X. malinche* reference genome. **B**) Alignment using haplotype 2 from the *X. malinche* reference genome. *X. malinche* is fixed for the non-false gravid spot phenotype. Both haplotypes are completely co-linear with the non-false gravid spot *X. birchmanni* haplotype. **C**) Alignment of *X. hellerii*, a southern swordtail species ~4-5 million years diverged from *X. birchmanni* is also largely co-linear with the non-false gravid spot haplotype in *X. birchmanni*. *X. hellerii*, as well as the entire southern swordtail clade, lack the false gravid spot polymorphism.

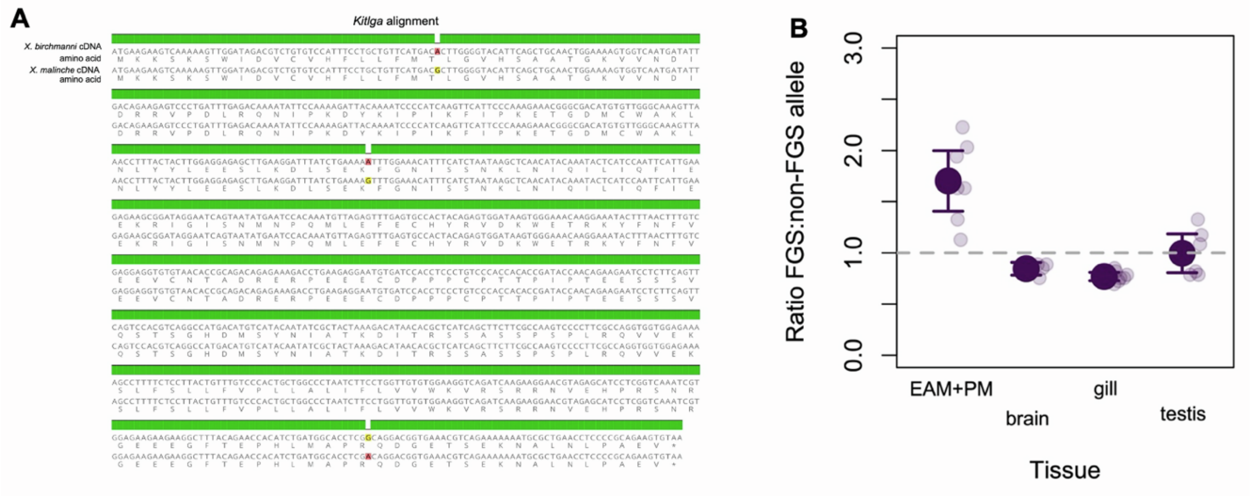

Figure S9. Allele-specific expression analysis, related to Figure 3. A) Alignment of *kitlga* between *X. birchmanni* and *X. malinche* cDNA and amino acid sequences. Despite having no nonsynonymous substitutions, there are several fixed synonymous differences in the *kitlga* cDNA between *X. birchmanni* and *X. malinche.* These differences were used to design allele-specific expression primers. **B**) Results from a second pyrosequencing primer targeting cDNA site 57 in *kitlga* confirm results presented in the main text that the false gravid spot allele is associated with higher tissue and allele specific expression in the EAM+PM tissue. The ratio of expression attributable to the false-gravid (FGS) versus non-false gravid (non-FGS) alleles is plotted on the y-axis. This suggests the expression differences in *kitlga* are under *cis*-regulatory control. Expression is normalized to the *X. malinche* allele. Large points and whiskers denote mean ± 2 standard errors, and small points represent individual results. Results shown in main text Figure 3 target cDNA site 759 in *kitlga*, which had slightly better performance in parental test samples.

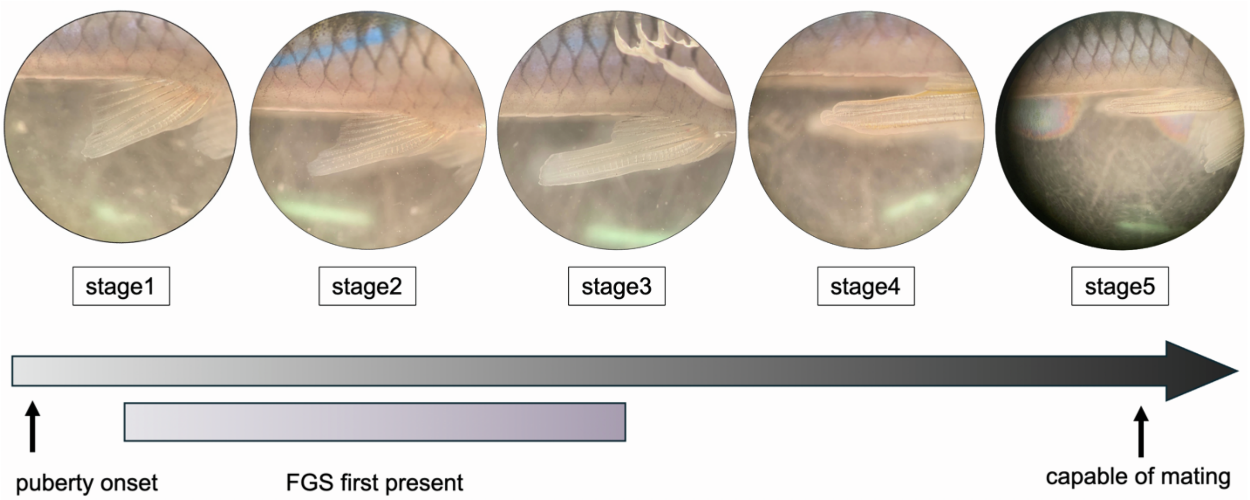

Figure S10. Developmental staging of *X. birchmanni* juveniles, related to Figure 3. Example images of gonopodial differentiation taken from the developmental time-tracking experiment. This experiment was used to determine when during gonopodial differentiation the false gravid spot phenotype developed. Males typically developed the false gravid spot phenotype between stages 1 and 3 of gonopodial development (indicated by the purple bar) but are not capable of mating until stage 5, when the hook and spikes form on the gonopodium and the cuticle recedes.

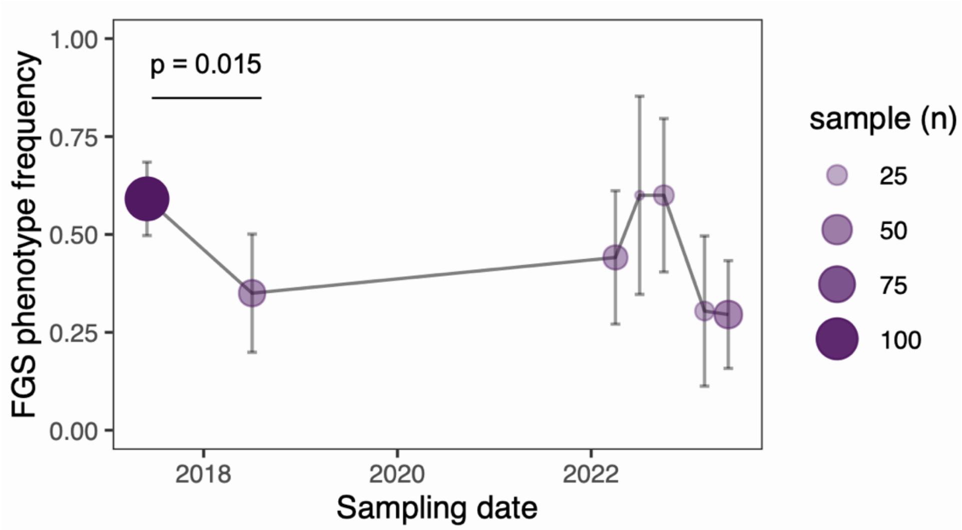

Figure S11. False gravid spot frequency across time at Coacuilco, related to Figure 4. Phenotypic frequencies of the false gravid spot (FGS) at Coacuilco fluctuate over time. Point size and opacity is proportional to the number of males collected at that time point. Error bars denote ± 2 binomial standard errors. Frequencies significantly differed between 2017 and 2018 (two proportion Z-test, $p=0.015$).

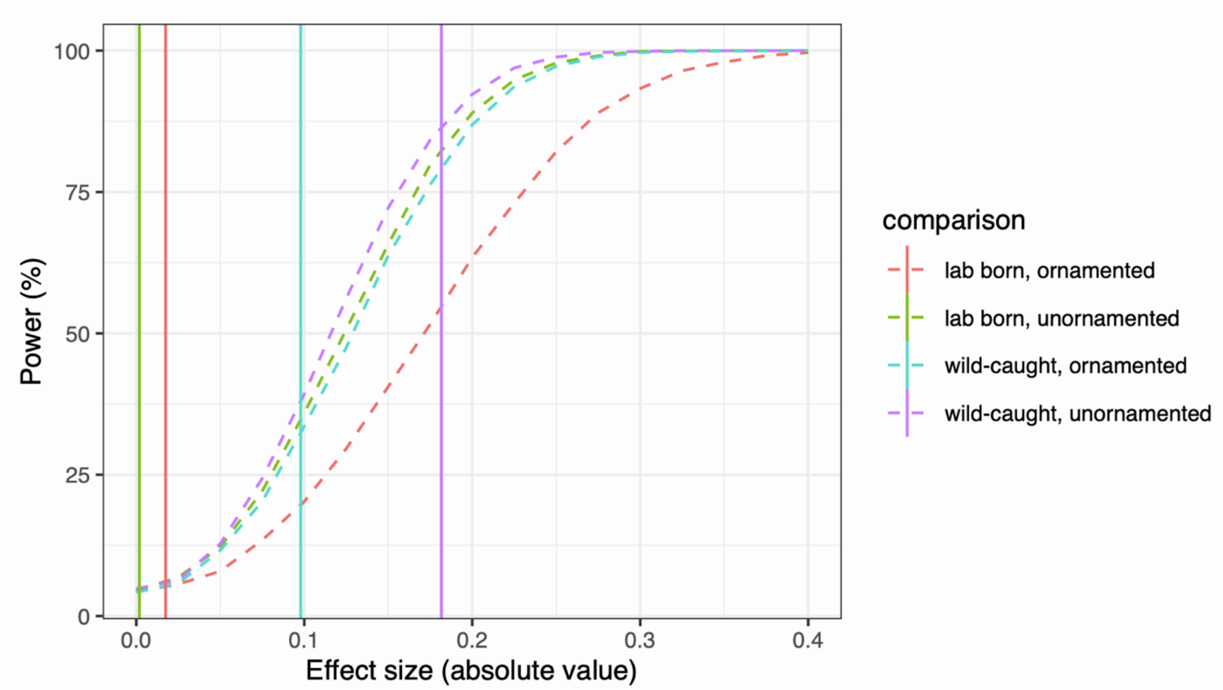

Figure S12. Expected power to detect a true effect of varying strength in behavioral trials, related to Figure 5. Results of simulations for each female group and animation stimulus under scenarios with known effect sizes for female preference. Empirically inferred effect sizes (mean difference in the proportion of the trial spent associated with each stimulus) from experimental data are shown with vertical dashed lines. This analysis revealed that we expect to have similar power for 3 of the 4 experiments, with lab-born females tested against ornamented stimuli experiment having lower expected power than the other groups. As expected, our power increased when effect sizes were larger, exceeding 80% in most groups at an effect size of ~0.175. This suggests that for experiments with large effect sizes, such as those we observe in trials of wild-caught females tested against unornamented stimuli, we have excellent power to detect preference. However, at somewhat weaker effect sizes, we expect to have poor power to detect an effect of interest. For example, we predict only ~35% power to detect an effect size of ~0.1. We note that this effect size is similar to the estimated effect from the wild-caught female preference experiment testing ornamented stimuli.

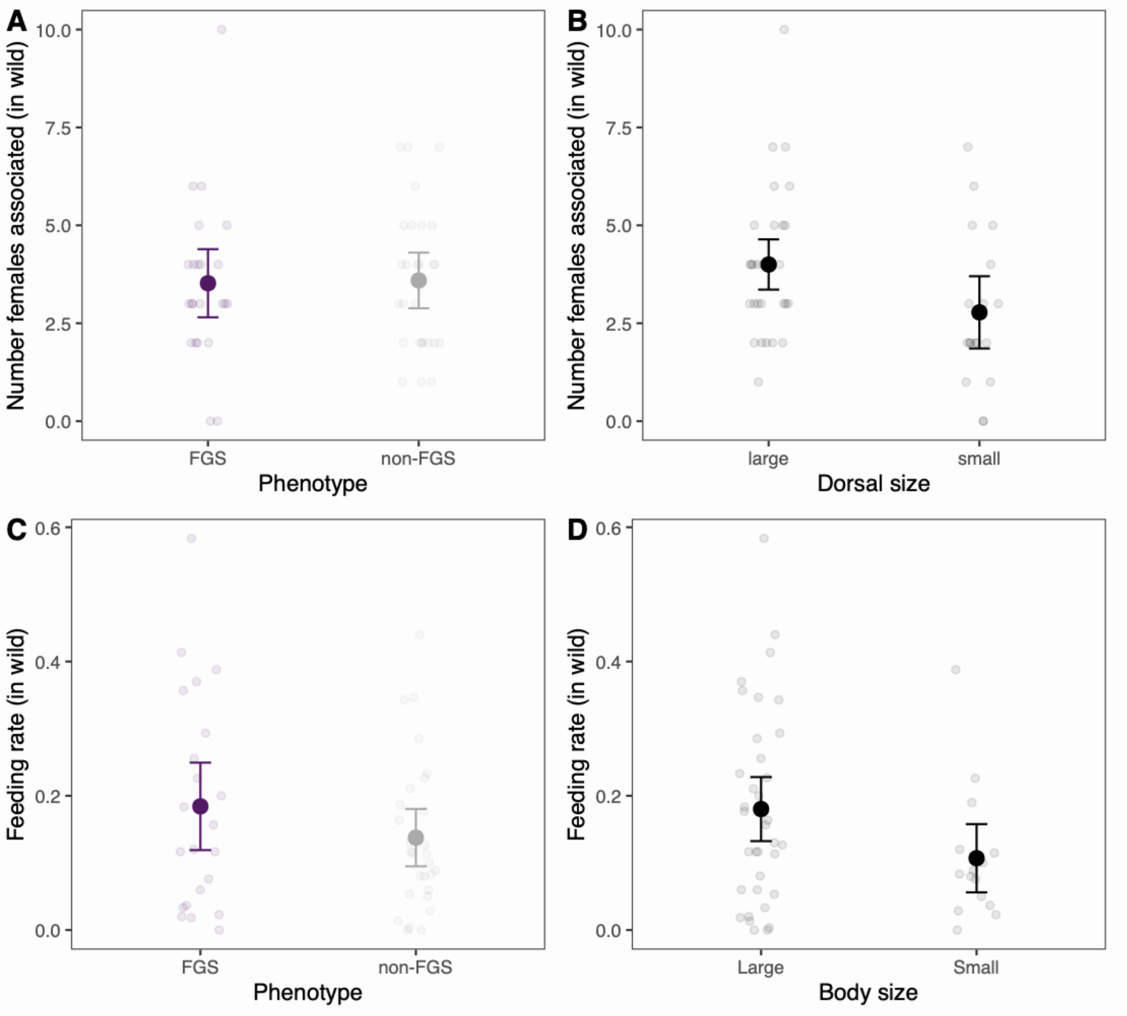

Figure S13. Results of additional behavioral observations of wild *X. birchmanni*, related to Figure 5. A) In observations of males in the Coacuilco population, males with false gravid spot (FGS) and without false gravid spot (non-FGS) did not differ in the number of females found within 1 m^2 (FGS variable excluded from the minimal GLM model). Dark points denote means and error bars indicate ± 2 standard errors. Light points indicate individual observations. B) Variation in the mean number of females found in association with the focal males was best explained by the size of a male's dorsal fin. Males with larger dorsal fins were typically found in association with more females. GLM: Likelihood ratio $\chi^2_1 = 4.9$, P = 0.027. C) In observations of males in the Coacuilco population, males with and without false gravid spot did not differ in their feeding rates (GLM: Likelihood ratio $\chi^2_1 = 2.3$, P = 0.132). FGS – false gravid spot males, non-FGS – non- false gravid spot males. D) Variation in male feeding rate (nips at substrate per minute) is best explained by male size, regardless of dorsal fin size or number of males in the vicinity (GLM: Likelihood ratio $\chi^2_1 = 4.4$, P = 0.036) Large body size was defined as males longer than a 4.4 cm and small body size was defined as males under 4.4 cm. This value was chosen because it corresponds to the mean male body size at Coacuilco,

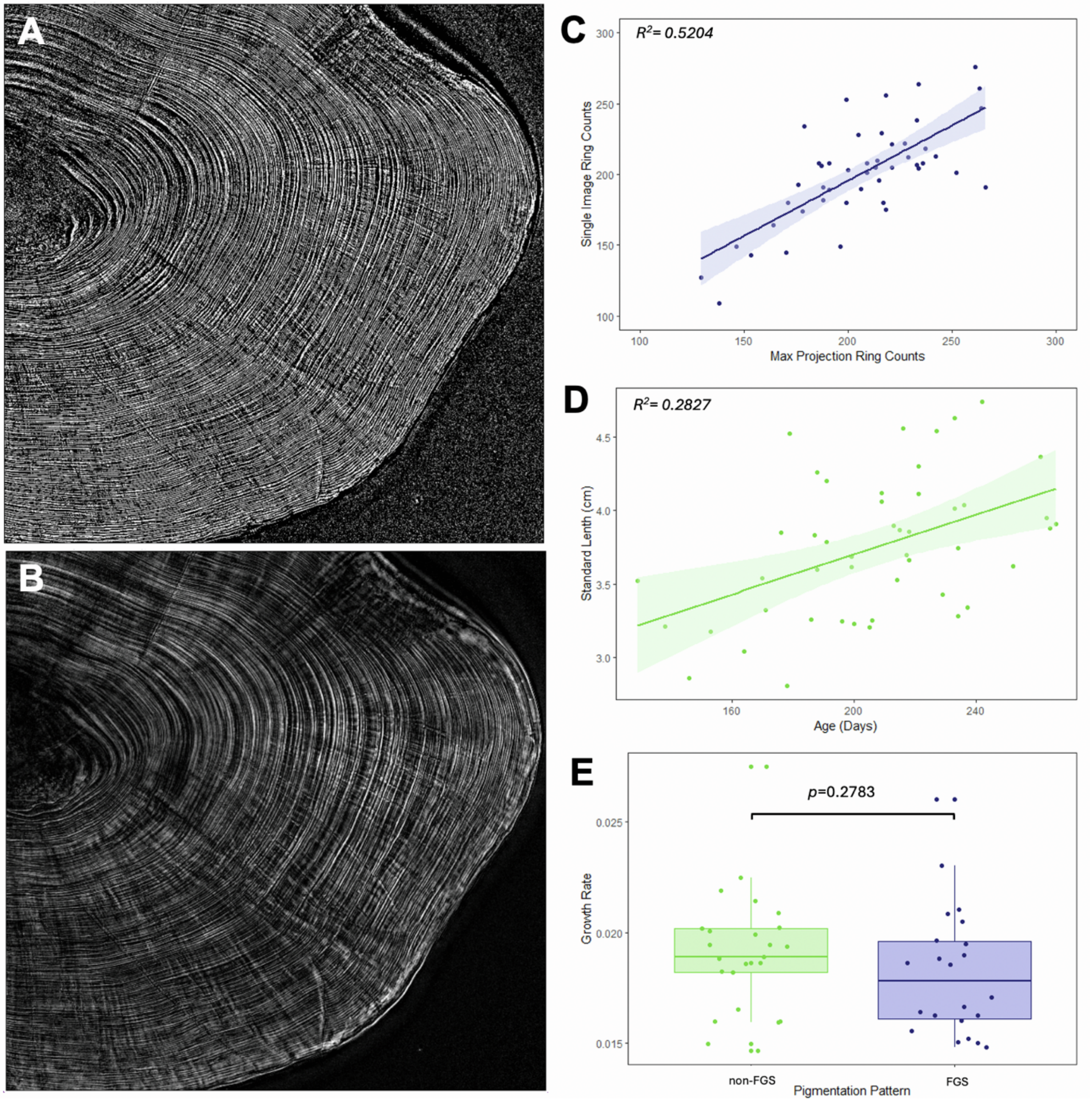

Figure S14. Otoliths revealed growth rates in wild caught males did not differ by phenotype, related to Figure 5. We post-processed the otolith image stacks in two ways, shown here. **A)** We compiled a max projection of each image stack and sharpened once and enhanced contrast by 0.35%. **B**) We also selected a single image from each stack and sharpened it once. Otolith rings were counted by 4 observers. **C**) Manual counts on max projection images of otoliths compared to single images yielded similar ring counts ($p<0.001$). However, the relationship was weaker than expected given that these results are technical replicates derived from counts from the same otolith. For subsequent analyses, the mean of the two counting methods was used. **D)** Male standard length is strongly associated with otolith ring count in our dataset ($p<0.0007$). This result is expected as males increase in size until sexual maturity, upon which growth and otolith deposition both slow dramatically. **E**) Juvenile male *X. birchmanni* with the false gravid spot (FGS) and fish without false gravid spot (non-FGS) do not have significantly different growth rates ($p<0.28$) in samples from natural populations. Growth rates were calculated by dividing standard length by otolith ring count (expected to be equivalent to age in days).

Species	*X. birchmanni*	*X. malinche*
**Sequencing technologies**	PacBio HiFi + Hi-C	PacBio HiFi + Hi-C
**Assembly size**	725.8 Mb	727.0 Mb
**Contig N50**	26.1 Mb	10.7 Mb
**Scaffold N50**	32.6 Mb	32.2 Mb
**Sequence in 24 largest scaffolds**	98.7%	98.4%
**Complete vertebrate BUSCOs**	98.7%	98.7%

Table S1. Contiguity and completeness metrics for chromosome-level reference assemblies for *X. birchmanni* and *X. malinche*, related to STAR Methods. Both reference assemblies are highly contiguous and complete. Individuals sequenced were both adult males without the false gravid spot phenotype. PacBio HiFi data for *X. birchmanni* were generated in this study, and for *X. malinche* were reassembled from Du *et al.*[S1]. Hi-C data for both species, which were used to scaffold contigs, were collected by Powell *et al.*[S2]. For these metrics, the Y chromosome alternate haplotype was excluded.

Description	Scientific Name	Max Score	Total Score	Query Cover	E value	Per. Ident	Acc. Len	Accession
PREDICTED: *Xiphophorus couchianus* piggyBac transposable element-derived protein 4-like (LOC114160923), mRNA	*Xiphophorus couchianus*	3302	4050	0.44	0	0.9913	2075	XM_028043852.1
PREDICTED: *Xiphophorus hellerii* piggyBac transposable element-derived protein 4-like (LOC116730547), mRNA	*Xiphophorus hellerii*	3103	3388	0.39	0	0.9713	2000	XM_032579869.1
PREDICTED: *Xiphophorus couchianus* piggyBac transposable element-derived protein 4-like (LOC114134966), mRNA	*Xiphophorus couchianus*	2968	3716	0.4	0	0.9957	1868	XM_028001868.1
PREDICTED: *Xiphophorus couchianus* piggyBac transposable element-derived protein 4-like (LOC114144083), mRNA	*Xiphophorus couchianus*	2931	3673	0.4	0	0.9914	1869	XM_028016557.1
PREDICTED: *Xiphophorus couchianus* piggyBac transposable element-derived protein 4-like (LOC114145772), mRNA	*Xiphophorus couchianus*	2771	3796	0.41	0	0.9916	1921	XM_028019331.1

Table S2. The insertion within the complex structural variant is a piggyBac transposable element, related to Figure 2. Top five blast hits for insertion implicates a Xiphophorus piggyBac protein 4-like transposable element. The database queried was the Standard Nucleotide Database.

Gene id	log2FoldChange	p-value	Gene symbol	tissue
g22533	3.726	7.61E-45	PMEL	PM+EAM
g5347	4.104	2.68E-42	tyrp1	PM+EAM
g15929	3.267	2.71E-34	TYRP1	PM+EAM
g9067	3.276	5.66E-31	MLANA	PM+EAM
g17743	3.072	1.03E-29	mreg	PM+EAM
g13517	2.712	6.66E-21	SLC2A11	PM+EAM
g10491	2.897	3.98E-18	Oca2	PM+EAM
**g2346**	**1.993**	**1.24E-12**	**kitlga**	**PM+EAM**
g15639	3.876	2.30E-12	-	PM+EAM
g20179	2.785	2.30E-12	tyr	PM+EAM
g17988	2.253	9.06E-12	Tspan10	PM+EAM
g16685	2.197	1.24E-10	CKB	PM+EAM
g10565	1.752	3.31E-10	-	PM+EAM
g4660	2.479	9.26E-10	slc24a5	PM+EAM
g25700	2.002	3.08E-08	ZDHHC2	PM+EAM
g10070	2.265	7.78E-07	ATCAY	PM+EAM
g23055	2.23	4.19E-06	Kif5b	PM+EAM
g14511	1.673	7.58E-06	slc30a8	PM+EAM
g1977	1.634	8.81E-06	DHFR	PM+EAM
g12495	1.724	1.24E-05	tyr	PM+EAM
g10340	0.696	1.40E-05	PRDX6	PM+EAM
g18049	4.135	1.74E-05	cyp2k1	PM+EAM
g4237	4.488	1.87E-05	Prss2	PM+EAM
g7785	1.871	1.87E-05	Fgf14	PM+EAM
g9181	2.234	4.69E-05	Slc45a2	PM+EAM
g13239	1.955	5.79E-05	adra2b	PM+EAM
g18048	2.86	7.02E-05	cyp2k4	PM+EAM
g15620	1.273	1.50E-04	Fcgr1	PM+EAM
g17347	1.544	1.50E-04	OPN5	PM+EAM
g24450	1.821	2.07E-04	Hmx2	PM+EAM
g24576	1.141	2.78E-04	Arhgef33	PM+EAM
g16256	2.239	3.17E-04	-	PM+EAM
g25605	1.604	6.37E-04	MCHR2	PM+EAM
g13516	1.795	6.82E-04	SLC2A11	PM+EAM
g25329	2.117	8.28E-04	SLC43A3	PM+EAM
g13112	1.093	8.97E-04	Rab27b	PM+EAM
g26355	0.917	1.41E-03	MLPH	PM+EAM
g16159	3.408	1.57E-03	Cdon	PM+EAM
g20326	1.35	2.58E-03	SLC30A1	PM+EAM
g26404	0.342	3.24E-03	DYNLT3	PM+EAM

g1653	0.511	3.67E-03	FAM180B	PM+EAM
g14087	0.335	3.83E-03	C1orf216	PM+EAM
g20182	1.546	3.83E-03	Rab38	PM+EAM
g9068	0.641	3.98E-03	KIAA2026	PM+EAM
g8175	-0.619	4.24E-03	MXRA5	PM+EAM
g25419	1.609	4.68E-03	BSCL2	PM+EAM
g18014	0.534	4.97E-03	lgals3bpb	PM+EAM
g8003	1.823	6.20E-03	DCT	PM+EAM
g18018	2.548	7.08E-03	-	PM+EAM
g812	1.747	7.17E-03	DTNBP1	PM+EAM
g21055	2.186	7.89E-03	SLC10A1	PM+EAM
g17804	0.844	8.93E-03	Bax	PM+EAM
g6856	-0.864	8.93E-03	Znf521	PM+EAM
g1706	1.171	1.01E-02	Trpm1	PM+EAM
g14199	0.916	1.21E-02	-	PM+EAM
g15623	1.942	1.21E-02	CD22	PM+EAM
g1094	0.246	1.21E-02	ada	PM+EAM
g26406	0.735	1.38E-02	GHR	PM+EAM
g17068	0.911	1.39E-02	Pde7b	PM+EAM
g14210	-1.967	1.52E-02	Mr1	PM+EAM
g5820	1.123	1.71E-02	-	PM+EAM
g17171	-1.286	2.09E-02	HMGXB3	PM+EAM
g2876	-1.286	2.09E-02	HMGXB3	PM+EAM
g6019	1.005	2.09E-02	-	PM+EAM
g15560	-1.092	2.17E-02	GIMAP7	PM+EAM
g10805	-1.654	2.18E-02	SYT2	PM+EAM
g898	0.325	2.18E-02	Cd300ld3	PM+EAM
g23276	-0.254	2.43E-02	EMILIN2	PM+EAM
g10425	-1.419	2.67E-02	-	PM+EAM
g22864	1.201	2.67E-02	LDLRAD2	PM+EAM
g1330	0.268	2.86E-02	-	PM+EAM
g3380	0.442	3.13E-02	C4	PM+EAM
g18578	0.121	3.27E-02	MYH9	PM+EAM
g18249	1.302	3.61E-02	Ighv1-72	PM+EAM
g2290	-0.489	4.38E-02	MBL2	PM+EAM
g18018	3.295	3.54E-08	-	Brain
g23218	-5.439	1.71E-07	Plcxd2	Brain
g15639	1.875	7.60E-07	-	Brain
g6314	0.383	1.37E-04	-	Brain
g7164	0.338	6.22E-04	PNP	Brain
g22141	-3.525	6.54E-04	-	Brain

g2290	-1.24	7.80E-04	MBL2	Brain
g6827	0.485	2.16E-03	tf	Brain
g2091	0.322	2.53E-03	Lamb1	Brain
g4681	0.32	3.78E-03	Mfge8	Brain
g11984	0.323	4.48E-03	slc22a6	Brain
g9930	0.459	4.48E-03	Slc5a5	Brain
g2312	-0.371	4.66E-03	Snupn	Brain
g12401	0.274	8.95E-03	Trim16	Brain
g4490	0.771	8.97E-03	CILP	Brain
g14076	-0.58	9.50E-03	MAP7D1	Brain
g3638	0.311	1.24E-02	Fxyd3	Brain
g24021	0.376	1.36E-02	-	Brain
g24663	1.735	1.42E-02	CDH1	Brain
g15633	0.491	1.66E-02	SIGLEC1	Brain
g12789	-0.413	2.09E-02	THAP9	Brain
g16159	1.243	2.10E-02	Cdon	Brain
g6936	0.244	2.23E-02	Higd1a	Brain
g20934	0.817	2.26E-02	TY3B-G	Brain
g7711	0.202	2.31E-02	LONRF2	Brain
g113	0.214	2.70E-02	-	Brain
g2551	0.73	2.79E-02	TG	Brain
g7165	0.304	2.79E-02	KCNJ10	Brain
g9980	-0.292	2.79E-02	ATP13A3	Brain
g21989	0.291	2.83E-02	-	Brain
g2955	-0.437	3.45E-02	-	Brain
g19988	0.249	3.50E-02	apoa1	Brain
g7684	0.208	4.12E-02	COX17	Brain
g17650	0.552	4.55E-02	CLDN4	Brain
g15006	0.248	4.89E-02	Slc6a19	Brain
g23840	-0.26	4.89E-02	-	Brain
g15777	0.252	4.89E-02	Plac8l1	Brain
g16101	-0.474	4.99E-02	-	Brain

Table S3. Results of DESeq2 analysis of differential expression across several tissues, related to Figure 3. Genes are sorted by p-value, with corrected p-values less than 0.05 included in the table. Differentially expressed genes between false gravid spot and non-false gravid spot males in the pigmented PM+EAM tissue include *kitlga*, and several other melanophore marker genes, while no such pattern emerges for brain tissue.

Primer target	Forward primer	Reverse primer	Sequencing primer	Performance in pure parentals
Kitlga site 57	TGGAGAAGAAGAAGGCTTTACAG	O-GAGGTTCAGCGCATTTTTTT	TCTGATGGCACCTCG	97-99%
Kitlga site 759	O-TCCATTTCCTGCTGTTCATGAC	TTTTCCAGTTGCAGCTGAATGTAC	GCTGAATGTACCCCA	98-99%

Table S4. Primers used in pyrosequencing quantification of allele specific expression of *kitlga*, related to STAR Methods. Primers were designed with the Qiagen Pyromark software. Performance in pure parentals refers to the estimated allelic support for the species-specific allele in two *X. birchmanni* and two *X. malinche* individuals.

Gonopodial Stage	Criteria
Stage 1 (onset of external puberty phenotype)	• Ray 3 thickens<br>• Fin has definite acute angle at cephalodistal corner<br>• Rays 3,4,5 short<br>• Ray 4 longer than 3 and 5<br>• Rays 3-4-5 form a fleshy protuberance at end of fin
Stage 2	• Rays 3-4-5 elongate beyond margin of fin<br>• Segments of ray 3 increase from 9 to 22
Stage 3	• Proximal spines differentiate on ray 3 (closer to body)
Stage 4	• Distal serrae form on back half of ray 4
Stage 5 (sexually mature)	• Blade appears at end of ray 3<br>• Gonopodium stiffens<br>• Skin shrinks, ends of spines flush with periphery<br>• Tips of proximal spines visible

Table S5. Male tracking maturation criteria, related to Figure 3. Criteria used to classify gonopodial differentiation into stages 1-5 for male developmental staging experiment. Based on Kallman and Schreibman[S3].

Supplementary References

S1. Du, K., Ricci, J.M.B., Lu, Y., Garcia-Olazabal, M., Walter, R.B., Warren, W.C., Dodge, T.O., Schumer, M., Park, H., Meyer, A., et al. (2024). Phylogenomic analyses of all species of swordtail fishes (genus *Xiphophorus*) show that hybridization preceded speciation. Nat. Commun. *15*, 6609. 10.1038/s41467-024-50852-6.

S2. Powell, D.L., García-Olazábal, M., Keegan, M., Reilly, P., Du, K., Díaz-Loyo, A.P., Banerjee, S., Blakkan, D., Reich, D., Andolfatto, P., et al. (2020). Natural hybridization reveals incompatible alleles that cause melanoma in swordtail fish. Science *368*, 731–736. 10.1126/science.aba5216.

S3. Kallman, K.D., and Schreibman, M.P. (1973). A sex-linked gene controlling gonadotrop differentiation and its significance in determining the age of sexual maturation and size of the platyfish, *Xiphophorus maculatus*. Gen. Comp. Endocrinol. *21*, 287–304. 10.1016/0016-6480(73)90061-0.
